# Further studies on the *Pselaphodes* complex of genera from China (Coleoptera, Staphylinidae, Pselaphinae)

**DOI:** 10.3897/zookeys.275.4571

**Published:** 2013-03-04

**Authors:** Zi-Wei Yin, Peter Hlaváč, Li-Zhen Li

**Affiliations:** 1 Department of Biology, College of Life and Environmental Sciences, Shanghai Normal University, 100 Guilin Road, Shanghai, 200234, P. R. China; 2 Czech University of Life Sciences, Faculty of Forestry and Wood Sciences, Department of Forest Protection and Game Management , Kamýcká 1176, CZ-165 21 Praha 6-Suchdol, Czech Republic

**Keywords:** Staphylinidae, Pselaphinae, Tyrina, key, taxonomy, *Dayao*, *Labomimus*, *Pselaphodes*, China

## Abstract

New data on the *Pselaphodes* complex of genera (Pselaphitae: Tyrini) from China is presented. The generic limits of *Labomimus* Sharp and *Pselaphodes* Westwood are discussed and expanded. A revised key to the genera of the *Pselaphodes* complex is provided. New geographic evidence suggests that previously believed wide-spread species *Pselaphodes tianmuensis* Yin, Li & Zhao contains a number of related species, resulting in a division of the species to nine separate taxa. Fourteen new species belonging to three genera are diagnosed, described and illustrated: *Dayao emeiensis* Yin & Li, **sp. n**. (Sichuan), *Labomimus fimbriatus* Yin & Hlaváč, **sp. n.** (Yunnan), *Labomimus jizuensis* Yin & Hlaváč, **sp. n.** (Yunnan), *Labomimus simplicipalpus* Yin & Hlaváč, **sp. n.** (Sichuan), *Pselaphodes anhuianus* Yin & Li, **sp. n.** (Anhui), *Pselaphodes daii* Yin & Hlaváč, **sp. n.** (Sichuan), *Pselaphodes grebennikovi* Yin & Hlaváč, **sp. n.** (Yunnan), *Pselaphodes hainanensis* Yin & Li, **sp. n.** (Hainan), *Pselaphodes kuankuoshuiensis* Yin & Li, **sp. n.** (Guizhou), *Pselaphodes longilobus* Yin & Hlaváč, **sp. n.** (Hunbei, Yunnan), *Pselaphodes monoceros* Yin & Hlaváč, **sp. n.** (Xizang), *Pselaphodes pengi* Yin & Li, **sp. n.** (Sichuan), *Pselaphodes tiantongensis* Yin & Li, **sp. n.** (Zhejiang) and *Pselaphodes wrasei* Yin & Li, **sp. n.** (Yunnan). *Labomimus sichuanicus* Hlaváč, Nomura & Zhou (Sichuan) is redescribed and illustrated based on a paratype and the material from the type locality. Two recently described species, *Pselaphodes tibialis* Yin & Li (Yunnan), and *Pselaphodes venustus* Yin & Li (Yunnan), are transferred to *Labomimus* (**comb. n.**) due to the presence of a median metaventral fovea. New locality data is provided for *Pselaphodes aculeus* Yin, Li & Zhao (Anhui, Fujian, Guangxi, Hainan, Yunnan), *Pselaphodes maoershanus* Yin & Li (Guangxi, Guizhou), *Pselaphodes tianmuensis* (Zhejiang, Anhui, Fujian, Jiangxi, Guangxi) and *Pselaphodes pectinatus* Yin, Li & Zhao (Hainan), with the aedeagus newly illustrated for the latter species.

## Introduction

A large number of tyrine beetles (Staphylinidae: Pselaphinae: Tyrini) from China in various collections have been studied by the first author, with the cooperation of the second and third authors. The results of this study are a new concept of the *Pselaphodes* complex of genera, description of fourteen new species, two new combinations, and new locality data for four known species. We report this information herein.

### Material and methods

The material treated in this study is housed in the following public institutions and private collections:

**NSMT **National Museum of Nature and Science, Tokyo, Japan (Shûhei Nomura)

**SNUC** Insect Collection of Shanghai Normal University, Shanghai, China (Zi-Wei Yin)

**pcPH **private collection of Peter Hlaváč, Košice, Slovakia

**pcMS **private collection of Michael Schülke, Berlin, German

The collection data of the referred material are quoted verbatim. A slash (/) is used to separate lines on the same label, and a double slash (//) is used to separate different labels. Authors’ notes are included in ‘[]’. Type material bears the following type label: ‘HOLOTYPE [red] or PARATYPE [yellow] / [genus name, species name] / sp. n., [authors of the species] / det. 2013. The depository is indicated after the collection data of the respective species.

The terminological terms follow Chandler 2001, except for using ‘ventrite’ instead of ‘sternite’ when discussing the meso- and metathoracic structures.

All measurements are in millimeters. The following acronyms are applied: **AL**–length of the abdomen along the midline; **AW**–maximum width of the abdomen; **BL**–length of the body (= HL + PL + EL + AL); **EL**–length of the elytra along the sutural line; **EW**–maximum width of the elytra; **HL**–length of the head from the anterior clypeal margin to the occipital constriction; **HW**–width of the head across eyes; **PL**–length of the pronotum along the midline; **PW**–maximum width of the pronotum.

## Taxonomy

### *Pselaphodes* complex of genera (sensu Hlaváč 2002: 283)

#### Discussion.

The shape of maxillary palpomeres II–IV was usually used as an important character to separate genera of the *Pselaphodes* complex (Hlaváč 2002; Hlaváč and Chandler 2005). Use of the form of the maxillary palpi in combination with the foveal patterns, will usually lead to the recognition of most genera (Chandler 2001: 400). However, when more material of the homogeneous *Pselaphodes* complex of genera was studied, conflicts between these characters appeared, and some species cannot be assigned to any known genus based on their current definitions. One new species described here, e.g. *Labomimus simplicipalpus* sp. n., which has a well-defined setose median metaventral fovea (typical for *Labominus*), but small and completely symmetric palpomeres II–IV (typical for *Lasinus* and *Paralasinus*). Another species, described as *Pselaphodes monoceros* sp. n., has nearly symmetric maxillary palpi, with palpomeres III being indistinctly projecting laterally (*Pselaphodes* are usually with palpomeres II–IV strongly asymmetric), and has the male sexual character located on the frons (previously unknown in members of the complex). We do not believe there is a justification to erect any supraspecific taxa for these species; hence we here expand the generic limits of *Labomimus* and *Pselaphodes*. Consequently we provide a modified key to genera of the *Pselaphodes* complex.

#### Key to genera of *Pselaphodes* complex

(modified from Hlaváč 2002: 284)

**Table:** 

1	Second tarsomeres broadly lobed beneath the third and extending nearly to the tarsal claws	2
–	Second tarsomeres simple, linear, not strongly lobed, rarely extending beneath the third tarsomeres	3
2	Frontal foveae present; setose pronotal median and lateral antebasal foveae connected by shallow antebasal sulcus	*Taiwanophodes* Hlaváč
–	Frontal foveae absent; pronotum lacking antebasal sulcus, median antebasal fovea nude	*Nomuraius* Hlaváč
3	Setose median metaventral fovea present	4
–	Median metaventral fovea absent	6
4	Vertexal and frontal foveae absent or weakly-defined; head and pronotum roughly punctate; pronotal median longitudinal sulcus absent	*Linan* Hlaváč
–	Vertexal and frontal foveae well-defined; head and pronotum usually finely punctate; pronotal median longitudinal sulcus usually present	5
5	Pronotum lacking median antebasal fovea; elytra carinate	*Indophodes* Hlaváč
–	Pronotal median antebasal fovea well-defined; elytra not carinate	*Labomimus* Sharp
6	Maxillary palpi with palpomeres II–IV simple, completely symmetric	7
–	Maxillary palpi with at least some segment of II–IV asymmetric, roundly expanded or slightly to distinctly projecting laterally	8
7	Pronotum with antebasal sulcus connecting lateral antebasal foveae	*Paralasinus* Hlaváč & Nomura
–	Pronotum lacking antebasal sulcus	*Lasinus* Sharp
8	Head with frontal fovea	*Pselaphodes* Westwood
–	Head lacking frontal fovea	*Dayao* Yin, Li & Zhao

### Genus *Dayao* Yin, Li & Zhao

*Dayao* Yin, Li & Zhao, 2011b: 47. Type species: *Dayao pengzhongi* Yin, Li & Zhao, 2011b.

#### Dayao emeiensis

Yin & Li sp. n.

urn:lsid:zoobank.org:act:5647FE6D-0705-4361-8332-BD5ADF0A3D96

http://species-id.net/wiki/Dayao_emeiensis

Figs 1A 2

##### Type material 

(1 ♂, 1 ♀)**.** Holotype: ♂, labeled ‘CHINA: Sichuan, E’meishan City / E’mei Shan Mt., pass between / Xixiangchi and Yanwang Slope / 29°33'28"N, 103°20'36"E, 2200 m / (leaf litter, sifted), 2012.vii.23 / C. C. Dai, Z. Peng & Z. W. Yin leg.’ (SNUC). Paratype: ♀, same label data as holotype (SNUC).

##### Diagnosis.

Reddish brown; length 2.96; postgenae narrowed; antennomeres IX–XI enlarged, unmodified in both sexes; pronotum rounded at anterolateral margins; male with large metaventral processes; aedeagus with asymmetric median lobe.

##### Description.

**Figure 1.**
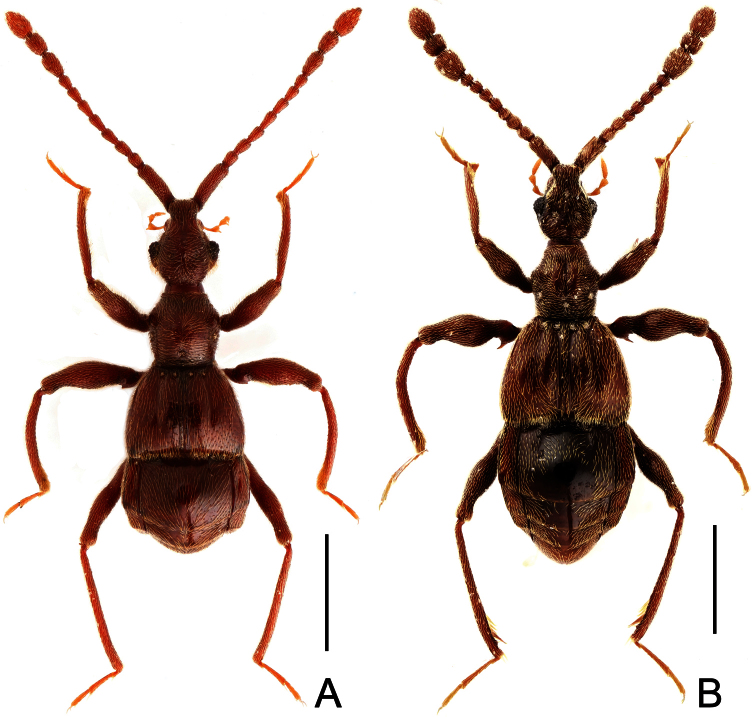
Male habitus of *Dayao emeiensis* (**A**) and *Labomimus fimbriatus* (**B**). Scales: 1.0 mm.

**Figure 2.**
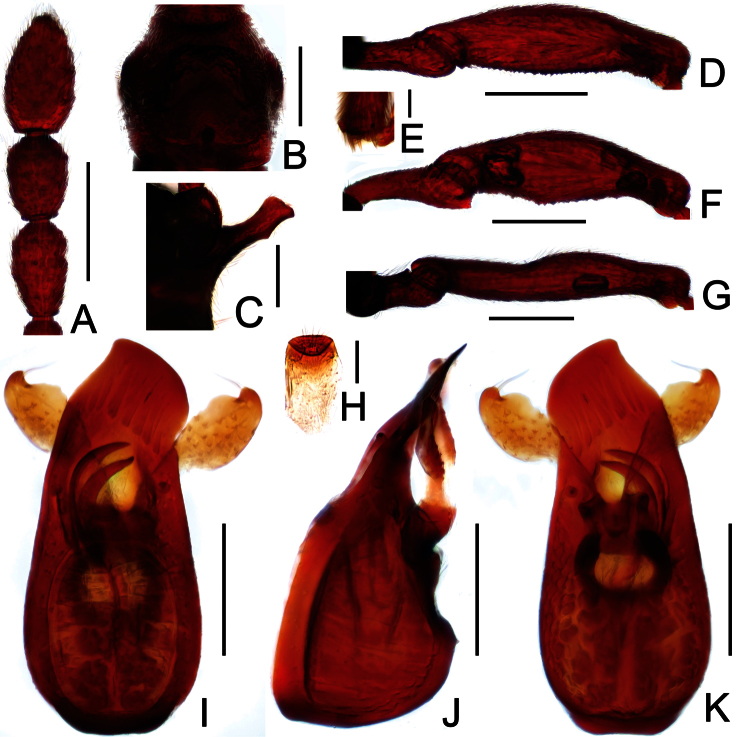
Diagnostic features of *Dayao emeiensis* in male. **A** antenna **B** pronotum **C** median metaventral process, in lateral view **D** protrochanter and profemur **E** apical portion of protibia **F** mesotrochanter and mesofemur **G** metatrochanter and metafemur **H** sternite IX **I** aedeagus, in dorsal view **J** same, in lateral view **K** same, in ventral view. Scales (mm): **A, B, D, F, G** = 0.3; **C, I, J, K** = 0.2; **H** = 0.1; **E** = 0.05.

Male (Fig. 1A). Length 2.96–2.97. Head longer than wide, HL 0.73, HW 0.62; eyes each composed of about 25 facets. Antennal clubs as in Fig. 2A. Pronotum (Fig. 2B) about as long as wide, PL 0.64, PW 0.62, rounded at anterolateral margins. Elytra wider than long, EL 0.73, EW 1.06. Long metaventral processes with truncate apices (Fig. 2C). Protrochanters and profemora simple (Fig. 2D); protibiae with small apical projection (Fig. 2E); mesotrochanters (Fig. 2F) slightly protuberant at ventral margin; metatrochanters and metafemora simple (Fig. 2G). Abdomen broad at base and narrowed apically, AL 0.86, AW 1.14. Sternite IX as in Fig. 2H. Aedeagus length 0.46, with symmetric median lobe broad (Figs 2I–K).

Female. Similar to male in general; BL 2.97, HL 0.74, HW 0.59, PL 0.65, PW 0.62, EL 0.68, EW 1.06, AL 0.90, AW 1.19. Eyes each composed of about 15 facets. Antennae simple; metaventral processes absent,.

##### Comparative notes.

Males of the new species can be readily separated from those of the only known congener, *Dayao pengzhongi*, by the unmodified antennae, the pronotum lacking tufts of long golden setae near the anterior margin, the much larger metaventral processes, and the aedeagus has broader parameres. *Dayao pengzhongi* has modified antennomeres IX and pronotum, and the aedeagus has relatively much thinner parameres (Yin et al. 2011b: 51, figs 11–13).

##### Distribution.

Southwest China: Sichuan.

##### Biology.

Adults were collected by sifting leaf litter in a mixed forest.

##### Etymology.

The new species is named after the type locality, E’mei Shan Mountain.

##### Notes. 

A female specimen (in pcPH) from Nibashan Mt., (Daxiangling Mts., ca. 50 km. W E’meishan) has slightly greater body size, and has each eye composed of about 20 facets. An associated male from Nibashan is needed for species identification.

### Genus *Labomimus* Sharp

*Labomimus* Sharp, 1883: 300. Type species: *Labomimus reitteri* Sharp, 1883.

#### Labomimus fimbriatus

Yin & Hlaváč sp. n.

urn:lsid:zoobank.org:act:2A8B615E-D062-4CEE-BA60-58D81342A3C1

http://species-id.net/wiki/Labomimus_fimbriatus

Figs 1B 3

##### Type material

(7 ♂♂, 6 ♀♀)**.** Holotype: ♂, labeled ‘CHINA: Yunnan, Lushui County / Pianma Town, Gaoligongshan Mt. / 25°58'46"N, 98°40'33"E, 3000 m, / (mixed leaf litter, sifted) / 2012.vi.24, Liang Tang leg. (SNUC). Paratypes: 1 ♂, same label data as holotype (SNUC); 2 ♂, 3 ♀♀, labeled ‘CHINA: Yunnan [CH07-24], Nujiang / Lisu Aut. Pref., Gaoligong Shan, valley 18 / km W Gongshan, 3020 m, 27°47'54"N, 98°30'13"E, mixed forest, litter, moss, / wood sifted, 7.VI.2007, M. Schülke’ (pcMS, SNUC); 1 ♀, CHINA: Yunnan [CH07-26], Nujiang / Lisu Aut. Pref., Gaoligong Shan, pass 21 / km NW Liuku, 3150 m, 25°58'22"N, 98°41'00"E, bamboo with shrubs, litter / sifted, 9.VI.2007, M. Schülke’ (pcMS); 1 ♀, same label data, except ‘25°58'49"N, 98°41'48"E’ (SNUC); 1 ♂, labeled ‘CHINA: Yunnan, Nujiang Lisu Pref., / Gaoligong Shan, “Cloud pass”, / 21 km NW Liuku, 25°58'21"N, 98°41'01"E, 3150 m, shrubs & / bamboo, litter sifted, 3.IX.2009, leg. M. Schülke [CH09-22a]’ (SNUC); 1 ♂, 1 ♀, same label data, except ‘2.IX.2009 D. W. Wrase [22A]’ (SNUC); 1 ♂, labeled ‘CHINA (Yunnan) / Nujiang Lisu Aut. Pref., / Gaoligong Shan, creek valley / 20 km NW Liuku, 3000 m, / 25°58'49"N, 98°41'48"E / (bamboo, shrub, litter sifted) / 9.VI.2007 D.W. Wrase [27]’ (pcMS).

##### Diagnosis.

Reddish brown; length 3.47–3.77; postgenae rounded laterally; antennomeres IX–XI enlarged; IX modified in male; pronotum roundly expanded at anterolateral margins; male with long curved metaventral processes; metacoxae simple; aedeagus with symmetric median lobe.

##### Description.

**Figure 3.**
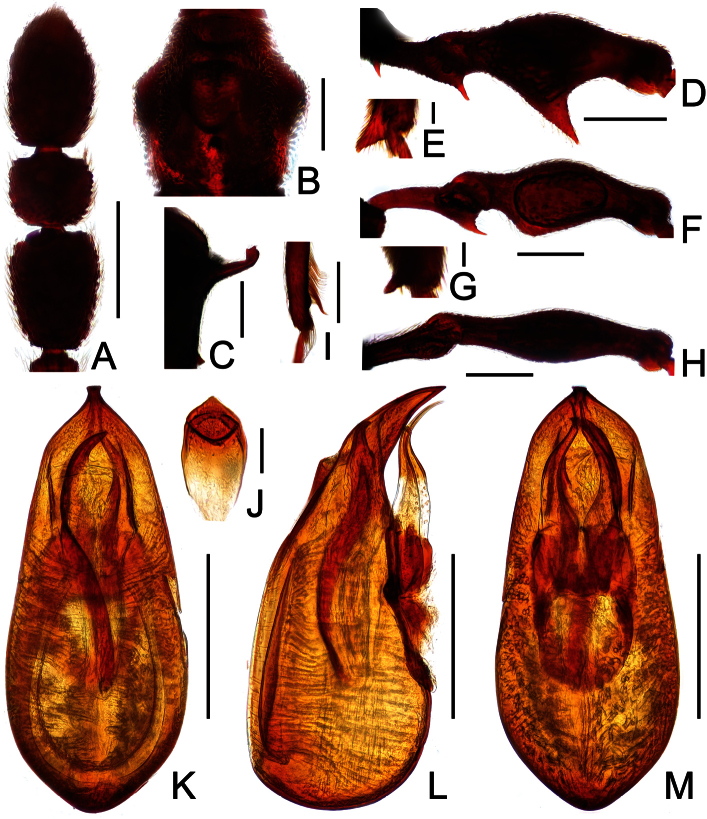
Diagnostic features of *Labomimus fimbriatus* in male. **A** antenna **B** pronotum **C** median metaventral process, in lateral view **D** procoxa, protrochanter and profemur **E** apical portion of protibia **F** mesotrochanter and mesofemur G apical portion of mesotibia **H** metatrochanter and metafemur **I** apical portion of metatibia **J** sternite IX **K** aedeagus, in dorsal view **L** same, in lateral view **M** same, in ventral view. Scales (mm): **A, B, C, D, F, H, I, K, L, M** = 0.3; **J** = 0.1; **E, G** = 0.05.

Male (Fig. 1B). Length 3.52–3.77. Head slightly longer than wide, HL 0.70–0.72, HW 0.60–0.65; eyes each composed of about 30 facets. Antennal clubs as in Fig. 3A. Pronotum (Fig. 3B) slightly longer than wide, PL 0.70–0.71, PW 0.65–0.69, roundly expanded at anterolateral margins. Elytra wider than long, EL 0.87–0.92, EW 1.22–1.26. Metaventral processes (Fig. 3C) long, curved anteriorly at apices. Procoxae, protrochanters and profemora spinose at ventral margin (Fig. 3D), protibiae with distinct triangular apical projection (Fig. 3E); mesotrochanters with large ventral spine, mesofemora roundly broadened ventrally (Fig. 3F), mesotibiae with small apical tubercle (Fig. 3G); metatrochanters and metafemora (Fig. 3H) simple, metatibiae with setose tuft near apices (Fig. 3I). Abdomen broad at base and narrowed apically, AL 1.25–1.42, AW 1.29–1.37. Sternite IX as in Fig. 3J. Aedeagus length 0.75, with symmetric median lobe (Figs 3K–M).

Female. Similar to male in general; BL 3.47–3.65, HL 0.73–0.76, HW 0.62–0.63, PL 0.70–0.72, PW 0.64–0.65, EL 0.74–0.75, EW 1.25–1.32, AL 1.30–1.42, AW 1.37–1.47. Eyes each composed of about 30 facets. Antennae not modified; metaventral processes absent.

##### Comparative notes.

This species is close to *Labomimus jizuensis* and *Labomimus simplicipalpus* (both described below) in sharing similar modifications of the antennae and legs. *Labomimus fimbriatus* and *Labomimus simplicipalpus* share a symmetric aedeagal median lobe. The two species can be separated by the larger size, nearly symmetric antennomeres X, and more slender aedeagus in *Labomimus fimbriatus*, while *Labomimus simplicipalpus* is much smaller in size, has strongly asymmetric antennomeres X, and the aedeagus is more robust. *Labomimus jizuensis* can be separated from both former species by the clearly asymmetric aedeagal median lobe.

##### Distribution.

Southwest China: Yunnan.

##### Biology.

Adults were commonly sifted from mixed leaf litter in shrubs and forests and are abundant in litter from appropriate habitats.

##### Etymology.

The Latin word ‘*fimbriatus*’ means ‘having a fringe, fringed’, referring to the fringed apical portion of the metatibiae of the new species.

#### Labomimus jizuensis

Yin & Hlaváč sp. n.

urn:lsid:zoobank.org:act:5A178599-49BF-4C09-8CB9-E45EE4CDA574

http://species-id.net/wiki/Labomimus_jizuensis

Figs 4A 5

##### Type material 

(3 ♂♂)**.** Holotype: ♂, labeled ‘CHINA: Yunnan / above Dali, 2700-2900 m / 14.IV.1999 / leg. W. SCHAWALLER’ (pcPH). Paratypes: 2 ♂♂, labeled ‘CHINA (Yunnan) Dali Bai Aut. / Pref., Jizu ShaN, path to cable car, 37 km NE Dali / 2450 m, 25°58'N, 100°23'E / (mixed forest, litter, moss sifted) / 5.IX.2009 D.W. Wrase [29]’ (pcMS, SNUC).

##### Diagnosis.

Reddish brown; length 3.54–3.64; postgenae rounded laterally; antennomeres IX–XI enlarged; IX–X modified in male; pronotum roundly expanded laterally at anterolateral margins; male with short metaventral processes; metacoxae simple; aedeagus with asymmetric median lobe.

##### Description.

**Figure 4.**
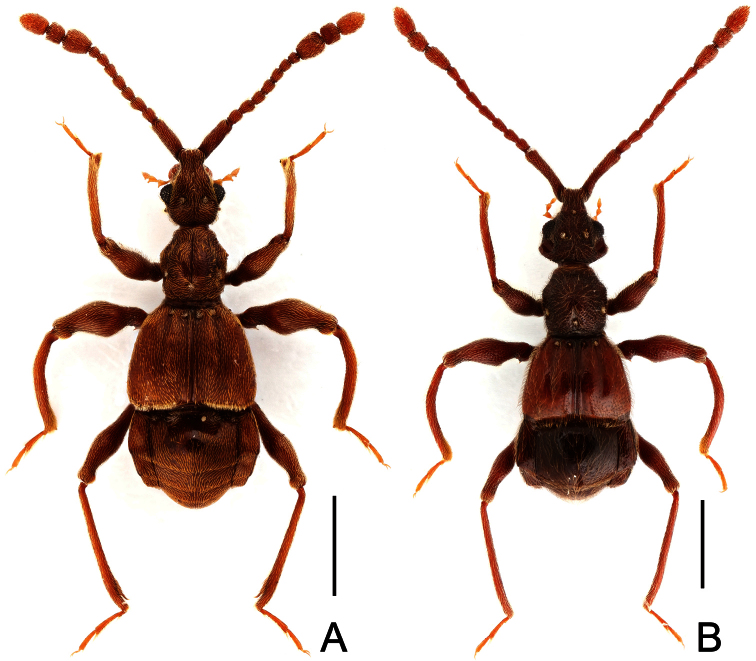
Male habitus of *Labomimus jizuensis* (**A**) and *Labomimus sichuanicus* (**B**). Scales: 1.0 mm.

**Figure 5.**
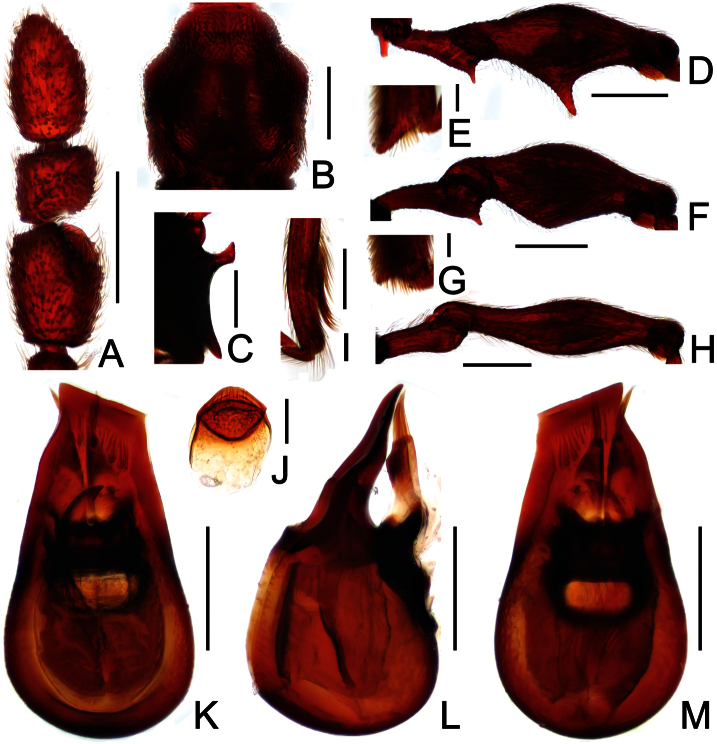
Diagnostic features of *Labomimus jizuensis* in male. **A** antenna **B** pronotum **C** median metaventral process, in lateral view **D** procoxa, protrochanter and profemur **E** apical portion of protibia **F** mesotrochanter and mesofemur G apical portion of mesotibia **H** metatrochanter and metafemur **I** apical portion of metatibia **J** sternite IX **K** aedeagus, in dorsal view **L** same, in lateral view **M** same, in ventral view. Scales (mm): **A, B, C, D, F, H** = 0.3; **I, K, L, M** =0.2; **J** = 0.1; **E, G** = 0.05.

Male (Fig. 4A). Length 3.54–3.64. Head longer than wide, HL 0.76–0.80, HW 0.63–0.65; eyes each composed of about 40 facets. Antennal clubs as in Fig. 5A. Pronotum (Fig. 5B) slightly longer than wide, PL 0. 0.73–0.76, PW 0.69–0.71, roundly expanded laterally at anterolateral margins. Elytra wider than long, EL 0.93–0.94, EW 1.29–1.31. Metaventral processes (Fig. 5C) short, truncate apically. Procoxae, protrochanters and profemora spinose at ventral margin (Fig. 5D), protibiae with distinct triangular apical projection (Fig. 5E); mesotrochanters with small ventral spine, mesofemora broadly thickened ventrally (Fig. 5F), mesotibiae with small apical tubercle (Fig. 5G); metatrochanters and metafemora (Fig. 5H) simple, metatibiae with setose tuft near apices (Fig. 5I). Abdomen broad at base and narrowed apically, AL 1.12–1.14, AW 1.32–1.38. Sternite IX as in Fig. 3J. Aedeagus length 0.57, with asymmetric median lobe (Figs 5K–M).

Female. Unknown.

##### Comparative notes.

*Labomimus jizuensis* is closely allied to *Labomimus fimbriatus* and *Labomimus simplicipalpus* as discussed above, it can be readily separated from both species by the clearly asymmetric aedeagal median lobe.

##### Distribution.

Southwest China: Yunnan.

##### Biology.

Individuals were collected by sifting litter and moss in mixed forests.

##### Etymology.

The new species is named after the locality where the two paratypes were collected, Jizushan Mountain.

#### Labomimus sichuanicus

Hlaváč, Nomura & Zhou

http://species-id.net/wiki/Labomimus_sichuanicus

Figs 4B 6

Labomimus sichuanicus Hlaváč, Nomura & Zhou, 2000: 149. Type locality: Qingchengshan Mountain, Sichuan, Southwest China.

##### Type material examined.

Paratype: ♂ [with aedeagus, tergite VIII and sternite VIII dissected, preserved in Canada balsam on a plastic plate pinned under the specimen], labeled ‘Wolong (1,770-1,790 m) / Wenchuan Xian / Sichuan Prov. // SE-China [should be SW-China] / 24.xi.1996, S. Nomura leg. // PARATYPE [blue] / *Labomimus sichuanicus* / Hlaváč, Nomura et Zhou’ (NSMT).

##### Other material examined

(5 ♂♂, 10 ♀♀)**.** 3 ♂♂, 9 ♀♀, labeled ‘CHINA: Sichuan, Dujiangyan City / Qingchengshan Mt., pass near / Baiyun Temple, 30°56'55"N, 103°28'28"E, 1650 m (bamboo / leaf, dead wood, sifted), 2012.vii.27 / C. C. Dai, Z. Peng & Z. W. Yin leg.’. 2 ♂♂, 1 ♀, same label data, except ‘1700 m’ (all SNUC).

##### Diagnosis.

Reddish brown; length 3.40–3.69; postgenae broadly expanded laterally; antennomeres IX–XI enlarged, simple in both sexes; pronotum rounded at anterolateral margins; male with short metaventral processes; metacoxae spinose ventrally; aedeagus with asymmetric median lobe.

##### Redescription.

**Figure 6.**
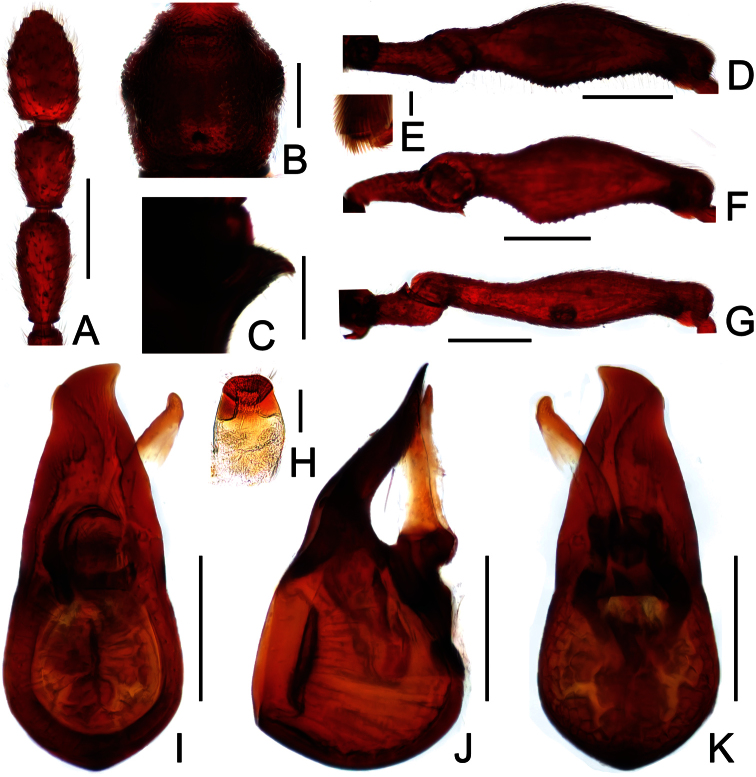
Diagnostic features of *Labomimus sichuanicus* in male. **A** antenna **B** pronotum **C** median metaventral process, in lateral view **D** protrochanter and profemur **E** apical portion of protibia **F** mesotrochanter and mesofemur **G** metacoxa, metatrochanter and metafemur **H** sternite IX **I** aedeagus, in dorsal view **J** same, in lateral view **K** same, in ventral view. Scales (mm): **A, B, D, F, G** = 0.3; **C, I, J, K** = 0.2; **H** = 0.1; **E** = 0.05.

Male (Fig. 4B). Length 3.40–3.50. Head longer than wide, HL 0.86–0.87, HW 0.68–0.72; eyes each composed of about 35 facets. Antennal clubs as in Fig. 6A. Pronotum (Fig. 6B) slightly longer than wide, PL 0.76–0.83, PW 0.74–0.75, rounded at anterolateral margins. Elytra wider than long, EL 0.86–0.87, EW 1.23–1.25. Metaventral processes very short (Fig. 6C). Protrochanters and profemora simple (Fig. 6D), protibiae with tiny apical spur (Fig. 6E); mesotrochanters with small ventral spine, mesofemora simple (Fig. 6F); metacoxae with short ventral protuberance, metatrochanters and metafemora simple (Fig. 6G). Abdomen broad at base and narrowed apically, AL 0.92–0.93, AW 1.31–1.38. Sternite IX as in Fig. 6H. Aedeagus length 0.55, with asymmetric median lobe elongate (Figs 3I–K).

Female. Similar to male in general; BL 3.59–3.69, HL 0.87–0.89, HW 0.64–0.70, PL 0.77–0.81, PW 0.73–0.77, EL 0.82–0.83, EW 1.32–1.34, AL 1.13–1.16, AW 1.43–1.47. Eyes each composed of about 28 facets. Metaventral processes absent.

##### Comparative notes.

This species is placed in the same group as *Labomimus shibatai* Sawada, *Labomimus dabashanus* Yin & Li, and *Labomimus schuelkei* Yin & Li by sharing the laterally expanded postgenae. *Labomimus sichuanicus* is closest to *Labomimus schuelkei* by sharing the postgenae being largely expanded laterally together with a thickened posterior margin, and the strongly elongate antennomeres V–VIII. The two species can be readily separated by the simple antennomeres IX–X, and the aedeagus with a much broader median lobe in *Labomimus sichuanicus*, while *Labomimus schuelkei* has strongly modified antennomeres IX–X, and the aedeagal median lobe is strongly narrowed dorsoventrally.

##### Distribution.

Southwest China: Sichuan.

##### Biology.

Individuals were sifted from mixed broad-leaved and bamboo leaf litter in a bush.

##### Remarks.

This species was described from three males and two females from Qingchengshan Mountain (type locality) and Wolong Natural Reserve of the Sichuan Province. The holotype and paratypes preserved in the Institute of Zoology, Academia Sinica, Beijing cannot be located at this time (Zhou per. comm.). The descriptions and illustrations provided by Hlaváč et al. (2000: 150) as well as a comparison with a paratype housed in NSMT leave no doubt that the material listed above is conspecific with the holotype.

#### Labomimus simplicipalpus

Yin & Hlaváč sp. n.

urn:lsid:zoobank.org:act:52556982-D4CF-45B5-9A08-BA9EFFE642A2

http://species-id.net/wiki/Labomimus_simplicipalpus

Figs 7A 8

##### Type material

(1 ♂)**.** Holotype: ♂, labeled ‘CHINA: Sichuan, Luding County / Hailuogou N. R., 28°35'47"N, 102°03'05"E, 2200–2300 m / (mixed leaf litter, sifted) / 2006.vii.27, Hu & Tang leg.’ (SNUC).

##### Diagnosis.

Reddish brown; length 3.00; postgenae rounded laterally; antennomeres IX–XI enlarged; IX–X modified in male; pronotum roundly expanded laterally; male with long metaventral processes; metacoxae simple; aedeagus with symmetric median lobe.

##### Description.

**Figure 7.**
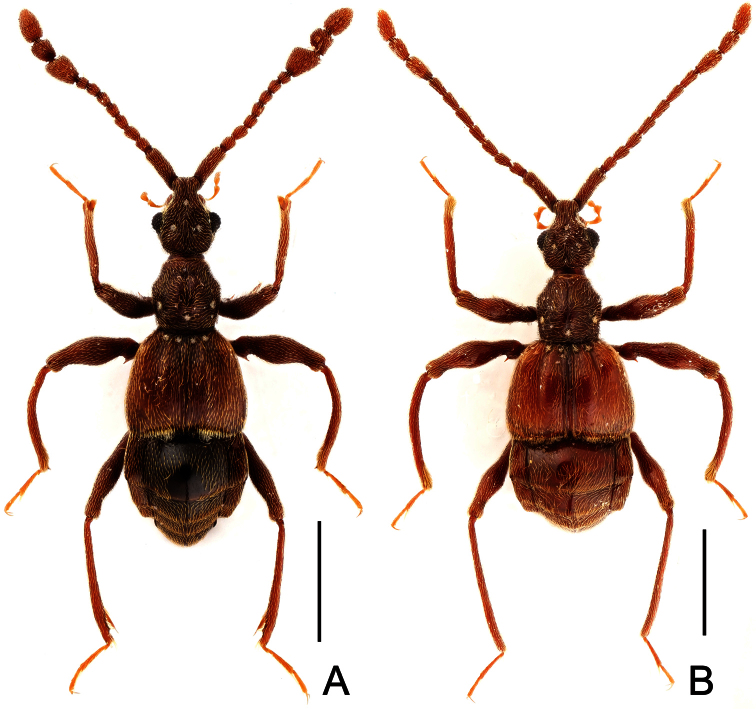
Male habitus of *Labomimus simplicipalpus* (**A**) and *Pselaphodes anhuianus* (**B**). Scales: 1.0 mm.

**Figure 8.**
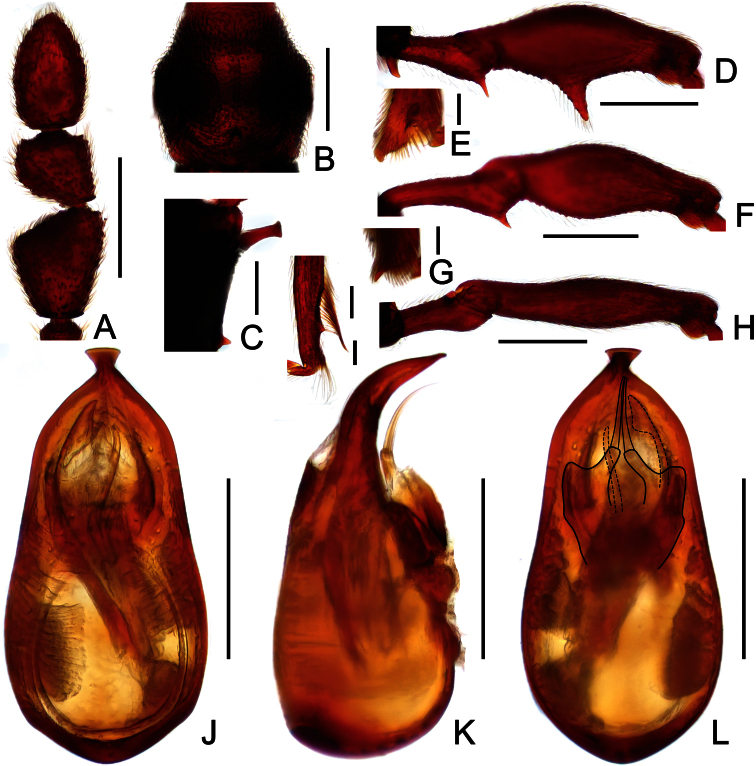
Diagnostic features of *Labomimus simplicipalpus* in male. **A** antenna **B** pronotum **C** median metaventral process, in lateral view **D** procoxa, protrochanter and profemur **E** apical portion of protibia **F** mesotrochanter and mesofemur **G** apical portion of mesotibia **H** metatrochanter and metafemur **I** apical portion of metatibia **J** aedeagus, in dorsal view **K** same, in lateral view **L** same, in ventral view. Scales (mm): **A, B, D, F, H** = 0.3; **C, J, K, L** = 0.2; **I** = 0.1; **E, G** = 0.05.

Male (Fig. 7A). Length 3.00. Head slightly longer than wide, HL 0.65, HW 0.59; eyes each composed of about 40 facets. Antennal clubs as in Fig. 8A. Pronotum (Fig. 8B) slightly longer than wide, PL 0.61, PW 0.59, roundly expanded laterally. Elytra wider than long, EL 0.81, EW 1.06. Metaventral processes (Fig. 8C) long, broadened and truncate at apices. Procoxae, protrochanters and profemora spinose at ventral margin (Fig. 8D), protibiae with distinct triangular apical projection (Fig. 8E); mesotrochanters with distinct ventral spine, mesofemora simple (Fig. 8F), mesotibiae with small apical tubercle (Fig. 8G); metatrochanters and metafemora (Fig. 8H) simple, metatibiae with setose tuft near apices (Fig. 8I). Abdomen broad at base and narrowed apically, AL 0.93, AW 1.06. Aedeagus length 0.45, with symmetric median lobe (Figs 3J–L).

Female. Unknown.

##### Comparative notes.

*Labomimus simplicipalpus* is closely related to *Labomimus fimbriatus* and *Labomimus jizuensis* as discussed above. The new species can be separated from *Labomimus jizuensis* by the symmetric aedeagal median lobe, from *Labomimus fimbriatus* by the smaller size, and the asymmetric antennomeres IX–X. The simple maxillary palpi of the new species are very unusual for *Labomimus*, and due to this the generic limit of *Labomimus* has to be expanded.

##### Distribution.

Southwest China: Sichuan.

##### Biology.

The single adult was collected from sifted mixed leaf litter in a forest.

##### Etymology.

The specific name refers to the simple maxillary palpi.

#### Labomimus tibialis

(Yin & Li) comb. n.

http://species-id.net/wiki/Labomimus_tibialis

Pselaphodes tibialis Yin & Li, 2012: 110. Type locality: Diancangshan Mountain, Dali, Yunnan, Southwest China.

##### Type material examined

(2 ♂♂)**.** Holotype: ♂, labeled ‘CHINA: Yunnan [CH07-09], / Dali Bai Aut. Pref., Diancang Shan 45 / km NW Dali, 2730 m, 26°01'20"N, 99°53'17"E, creek valley, pines, ferns, / sifted, 29.V.2007, M. Schülke’ (pcMS). Paratype: 1 ♂, same label data as holotype (pcMS).

##### Comments.

*Labomimus tibialis* is here transferred to *Labomimus* based on the presence of a median metaventral fovea. This species is placed in the same group as *Labomimus paratorus* Yin & Li, *Labomimus torus* (Yin, Li & Zhao), and *Labomimus venustus* (Yin & Li) based on the similar modifications of the male legs, and the strongly asymmetric aedeagal median lobe.

#### Labomimus venustus

(Yin & Li) comb. n.

http://species-id.net/wiki/Labomimus_venustus

Pselaphodes venustus Yin & Li, 2012: 111. Type locality: Jizushan Mountain, Dali, Yunnan, Southwest China.

##### Type material examined

(1 ♂, 1 ♀)**.** Holotype: ♂, labeled ‘CHINA (Yunnan) Dali Bai Aut. Pref., Jizu Shan, summit plateau, / 37 km NE Dali 3150 m, (mixed / forest, sifted from litter, moss) / 25°58'30"N, 100°21'36"E / 5.IX.2009 DW Wrase [28]’ (pcMS). Paratype: 1 ♀, same label data, except ‘leg. M. Schülke [CH09-28]’ (pcMS).

##### Comments.

*Labomimus venustus* is here transferred to *Labomimus* based on the presence of a median metaventral fovea. This species is placed in the group with *Labomimus paratorus* Yin & Li, *Labomimus tibialis* (Yin & Li), and *Labomimus torus* (Yin, Li & Zhao) based on the similar modifications of the male legs, and the strongly asymmetric aedeagal median lobe.

### Genus *Pselaphodes* Westwood

*Pselaphodes* Westwood, 1870: 129. Type species: *Pselaphodes villosus* Westwood, 1870

#### I. *Pselaphodes tianmuensis* species group

##### Included species.

Nine species are placed in the *tianmuensis*-group (here proposed), seven are described here as new: *Pselaphodes anhuianus* sp. n., *Pselaphodes daii* sp. n., *Pselaphodes hainanensis* sp. n., *Pselaphodes kuankuoshuiensis* sp. n., *Pselaphodes longilobus* sp. n., *Pselaphodes tianmuensis* Yin, Li & Zhao, *Pselaphodes tiantongensis* sp. n., *Pselaphodes wrasei* sp. n., *Pselaphodes yunnanicus* Hlaváč, Nomura & Zhou.

##### Diagnosis

(based on male features)**.** Medium to large in size (usually greater than 3 mm); apical three antennomeres enlarged; antennomeres IX slightly modified, with a disc-shaped process at apices, X–XI simple; protrochanters and profemora spinose at ventral margins; mesotrochanters usually with multiple ventral spines, mesofemora simple; metatrochanters and metafemora always simple; aedeagus with asymmetric median lobe, apical portion usually bent leftwards.

##### Discussion.

*Pselaphodes tianmuensis* Yin, Li & Zhao was recorded from a number of localities in China (Yin et al. 2010, 2011a). Putting aside the differences of aedeagal structure, populations from these localities present a relatively stable combination of male sexual characters; especially they share similar antennal modifications. Consequently, all of these were assigned to one, wide-spread species pending discovery of evidence leading to a different conclusion. Recently, when working on the material included in this paper, we found populations with two aedeagal forms that have a sympatric distribution (described as *Pselaphodes anhuianus* and *Pselaphodes longilobus* below). This geographical evidence proved not only the existence of two different species, but also the fact that other populations with different aedeagal forms cannot be treated as conspecific with *Pselaphodes tianmuensis*. Hence we reevaluate the specific limit of *Pselaphodes tianmuensis* and divide it into nine species.

Species identification of the group largely lies on the aedeagal from, the structure of the endophallus, the form of the metaventral processes, and the distribution. Further notes on these species, if any, will be provided in the ‘Comparative notes’ of the respective species.

##### Pselaphodes anhuianus

Yin & Li sp. n.

urn:lsid:zoobank.org:act:5B56F0E8-5E75-4AFD-B3A9-AA27055E010D

http://species-id.net/wiki/Pselaphodes_anhuianus

Figs 7B 9

###### Type material

(2 ♂♂, 1 ♀)**.** Holotype: ♂, labeled ‘CHINA: Anhui, Qianshan County / Tianzhu Shan National Park / 30°43'56"N, 116°27'11"E, 960 m / (mixed leaf litter, sifted) / 2006.iv.23, Hu & Tang leg.’ (SNUC). Paratypes: 1 ♀, same label data as holotype (SNUC); 1 ♂, labeled ‘P. R. CHINA, Hubei, Dabieshan, 31°06.013'N, 115°47.300'E / 11–21.vi.2008, 640 m / sifting, V. Grebennikov (pcPH).

###### Diagnosis.

Reddish brown; length 3.00–3.31; postgenae rounded laterally; antennomeres IX–XI enlarged; antennomeres IX modified in male; pronotum rounded at anterolateral margins; male with large metaventral processes; metacoxae simple; aedeagus with asymmetric median lobe.

###### Description.

**Figure 9.**
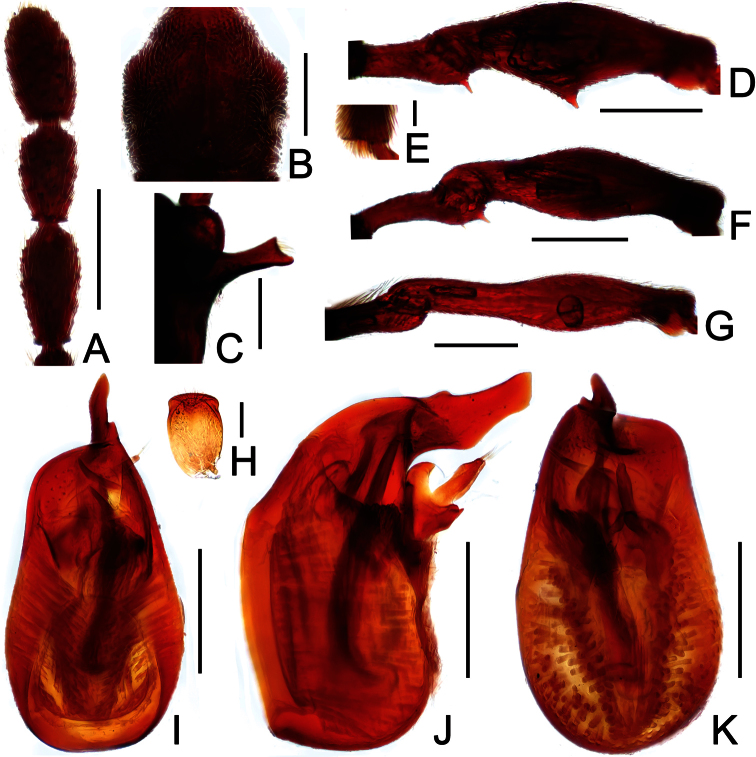
Diagnostic features of *Pselaphodes anhuianus* in male. **A** antenna **B** pronotum **C** median metaventral process, in lateral view **D** protrochanter and profemur **E** apical portion of protibia **F** mesotrochanter and mesofemur **G** metatrochanter and metafemur **H** sternite IX **I** aedeagus, in dorsal view **J** same, in lateral view **K** same, in ventral view. Scales (mm): **A, B, C, D, F, G** = 0.3; **I, J, K** = 0.2; **H** = 0.1; **E** = 0.05.

Male (Fig. 7B). Length 3.00–3.31. Head longer than wide, HL 0.66–0.72, HW 0.60–0.64; eyes each composed of about 50 facets. Antennal clubs as in Fig. 9A. Pronotum (Fig. 9B) slightly longer than wide, PL 0.65–0.72, PW 0.62–0.68, rounded at anterolateral margins. Elytra wider than long, EL 0.87–1.00, EW 1.25–1.34. Metaventral processes large, apically broadened (Fig. 9C). Protrochanters and profemora spinose ventrally (Fig. 9D), protibiae with tiny apical spur (Fig. 9E); mesotrochanters with small ventral spines, mesofemora simple (Fig. 9F); metatrochanters and metafemora simple (Fig. 9G). Abdomen broad at base and narrowed apically, AL 0.82–0.87, AW 1.20–1.31. Sternite IX as in Fig. 9H. Aedeagus length 0.60–0.66, with asymmetric median lobe (Figs 9I–K).

Female. Similar to male in general; BL 3.26, HL 0.72, HW 0.64, PL 0.71, PW 0.65, EL 0.87, EW 1.25, AL 0.96, AW 1.31. Eyes each composed of about 26 facets. Antennae simple; metaventral processes absent.

###### Comparative notes.

This species can be separated from the other members of the group primarily by the large, apically concaved metaventral processes, the more robust aedeagus with short apical portion of the median lobe, the structure of aedeagal endophallus, and its distribution.

###### Distribution.

East China: Anhui; Central China: Hubei.

###### Biology.

Adults were collected by sifting mixed leaf litter in forests.

###### Etymology.

The new species is named after the province where the type locality is located.

###### Notes.

Slight differences in body size and structure of the aedeagal endophallus were observed between specimens from Tianzhushan Mountain and Dabieshan Mountain. Since both localities belong to the Dabieshan Mountain Range, and all specimens were collected at low altitude (below 1000 m), the differences are considered to be intraspecific variation.

##### Pselaphodes daii

Yin & Hlaváč sp. n.

urn:lsid:zoobank.org:act:BD741270-7F89-410B-A1A0-E4DD02E87669

http://species-id.net/wiki/Pselaphodes_daii

Figs 10A 11

###### Type material

(19 ♂♂, 10 ♀♀)**.** Holotype: ♂, labeled ‘CHINA: Sichuan, Tianquan County / Er’lang Shan Mt., pass near summit / ca. 8 km SE Luding, 29°51'48"N, 102°17'32"E, 2800 m, (mixed leaf / litter, moss, sifted), 2012.vii.13 / C. C. Dai, Z. Peng & Z. W. Yin leg.’ (SNUC). Paratypes: 6 ♂♂, 5 ♀♀, same label data as holotype; 2 ♂♂, same label data, except ‘29°52'12"N, 102°17'03"E / 2700 m, 2012.vii.11’ (SNUC); 1 ♂, same label data, except ‘29°52’N, 102°18'E / 2900 m, 1999.VI.22 / leg. M. Schülke’ (pcMS); 2 ♂♂, labeled ‘P. R. CHINA, SichuaN, NE slope Gongga Shan / 29°48'15"N, 102°03'E / 44", 20.vi.2011, 2765 m / sift22. V. Grebennikov’ (pcPH, SNUC); 5 ♂♂, 2 ♀♀, same label data, except ‘29°50'50"N, 102°02'E / 28", 21.vi.2011, 3170 m / sift23’ (pcPH, SNUC); 1 ♂, 1 ♀ same label data, except ’18.VI.2011’ (pcPH, SNUC); 1 ♀, same label data, except ‘29°49'29"N, 102°03'E / 24', 2986 m, sift 25'’ (SNUC); 1 ♀, same label data, escept ‘29°52'10"N, 102°02'01"E / 12.VI.2011, 3620 m, sift16’ (pcPH); 1 ♂, labeled ‘P. R. CHINA, Sichuan / E slope Gongga Shan / 29°34'31"N, 102°00'E / 31", 23.vi.2011, 2832 m / sift26, V. Grebennikov’ (pcPH).

###### Other material examined.

1 ♂, labeled ‘CHINA: Sichuan, Luding County / Hailuogou N. R., 28°35'47"N, 102°03'05"E, 2200–2300 m / (mixed leaf litter, sifted) / 2006.vii.27, Hu & Tang leg.’ (SNUC).

###### Diagnosis.

Reddish brown; length 3.50–4.43; postgenae rounded laterally; antennomeres IX–XI enlarged; antennomeres IX modified in male; pronotum rounded at anterolateral margins; male with long, sharp metaventral processes; metacoxae simple; aedeagus with asymmetric median lobe.

###### Description.

**Figure 10.**
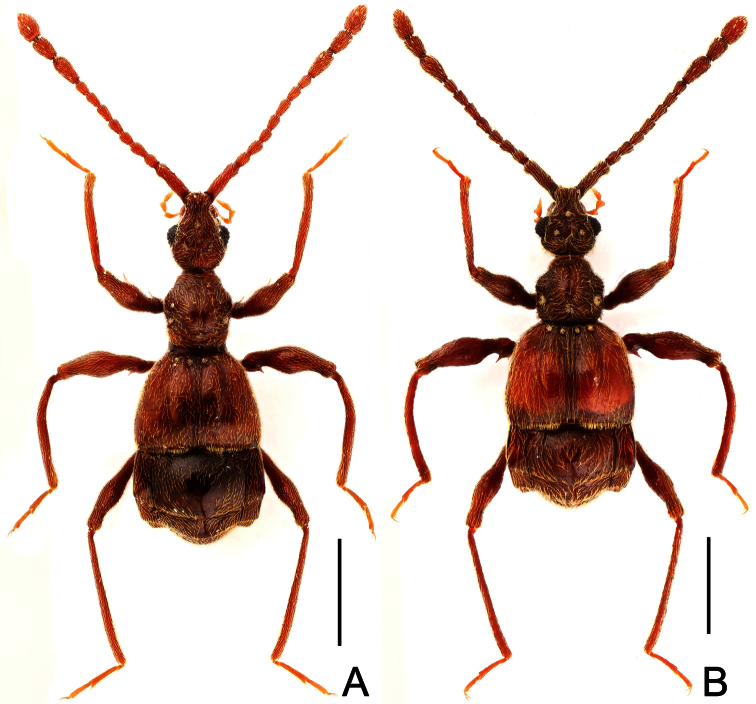
Male habitus of *Pselaphodes daii* (**A**) and *Pselaphodes hainanensis* (**B**). Scales: 1.0 mm.

**Figure 11.**
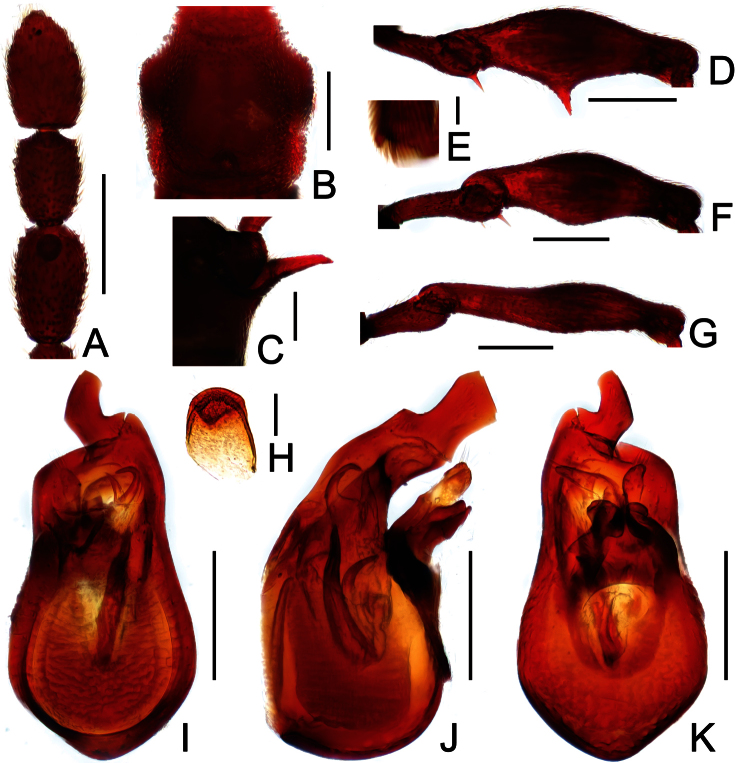
Diagnostic features of *Pselaphodes daii* in male. **A** antenna **B** pronotum **C** median metaventral process, in lateral view **D** protrochanter and profemur **E** apical portion of protibia **F** mesotrochanter and mesofemur **G** metatrochanter and metafemur **H** sternite IX **I** aedeagus, in dorsal view **J** same, in lateral view **K** same, in ventral view. Scales (mm): **A, B, D, F, G** = 0.3; **C, I, J, K** = 0.2; **H** = 0.1; **E** = 0.05.

Male (Fig. 10A). Length 3.50–3.76. Head longer than wide, HL 0.78–0.81, HW 0.62–0.65; eyes each composed of about 30 facets. Antennal clubs as in Fig. 11A. Pronotum (Fig. 11B) slightly longer than wide, PL 0.74–0.78, PW 0.66–0.69, rounded at anterolateral margins. Elytra wider than long, EL 0.86–0.92, EW 1.33–1.34. Metaventral processes long, apically narrowed (Fig. 11C). Protrochanters and profemora spinose ventrally (Fig. 11D), protibiae with tiny apical spur (Fig. 11E); mesotrochanters with small ventral spines, mesofemora simple (Fig. 11F); metatrochanters and metafemora simple (Fig. 11G). Abdomen broad at base and narrowed apically, AL 1.12–1.25, AW 1.40–1.46. Sternite IX as in Fig. 11H. Aedeagus length 0.61, with asymmetric median lobe (Figs 11I–K).

Female. Similar to male in general; BL 3.69–4.43, HL 0.77–0.84, HW 0.63–0.65, PL 0.76–0.81, PW 0.69–0.73, EL 0.88–0.89, EW 1.34–1.41, AL 1.28–1.89, AW 1.42–1.50. Eyes each composed of about 25 facets. Antennae unmodified; metaventral processes absent.

###### Comparative notes.

The new species can be separated from the other members of the group primarily by the long, sharp metaventral processes, the aedeagus with a short, apically truncate median lobe, the structure of aedeagal endophallus, and its distribution.

###### Distribution.

Southwest China: Sichuan.

###### Biology.

Adults were sifted from moss and mixed leaf litter in forests.

###### Etymology.

The new species is named after Cong-Chao Dai, co-collector of the type series.

###### Comments.

The single male from Hailuogou has the aedeagal endophallus being slightly different to the males from Er’langshan Mountain. Though this difference is currently considered to be intraspecific variation, we choose a conservative approach here and exclude this specimen from the type series.

##### Pselaphodes hainanensis

Yin & Li sp. n.

urn:lsid:zoobank.org:act:5815C2CE-AB0E-4C4C-B101-8DA5B653D4A6

http://species-id.net/wiki/Pselaphodes_hainanensis

Figs 10B 12

###### Type material

(24 ♂♂, 8 ♀♀)**.** Holotype: ♂, labeled ‘CHINA, Hainan, Baisha County / Yuanmeng, near Yinggezui Station / 19°03'10"N, 109°33'55E, 660 m / (mixed leaf litter, sifted) / 2011.iv.26, Wen-Xuan Bi leg.’ (SNUC). Paratypes: 7 ♂♂, same label data as holotype; 4 ♂♂, 1 ♀, labeled ‘China: Hainan Prov. / Wuzhishan Mt. / Guanshandian / 20.iv.2012, 500–700 m / Yin et al. leg.’; 1 ♂, same label data, except ’18.iv.2012, 650–700 m, Peng et al. leg.’; 1 ♂, 1 ♀, labeled ‘China: Hainan Prov. / Lingshui County / Diaoluoshan Mt. / 21.iv.2010 / alt. 1000 m / Yin Z. W. leg.’; 2 ♂♂, same label data, except ‘Light trap / 18.72510°N, 109.86861°E / 920 m, 2007.iii.25 / Shi H. L., Yuan F. coll.’; 1 ♂, 2 ♀♀, same label data, except ’26.iv.2012, 600–1000 m / Zi-Wei Yin leg.’; 3 ♂♂, same label data, except ‘Dai & Peng leg.’; 2 ♂♂, 3 ♀♀, labeled ‘China: Hainan Prov. / Ledong County / Jianfengling N. R. / alt. 1000 m, 15.IV.2012 / Ting Feng leg.’; 1 ♀, same label data, except ’16.IV.2012, 900 m / Yuan & Yin leg.’; 1 ♂, same label data, except ‘2.V.2012, Pan & Yin leg.’; 1 ♂, labeled ‘China: Hainan Prov. / Qiongzhong County / Limu Shan Mt. / Qijiacun, 650 m / 2010.IV.6 (light trap) // 19.17310°N, 109.71968°E / Mei-Yin Lin leg. [data in Chinese]’ (all SNUC).

###### Diagnosis.

Reddish brown; length 3.14–3.43; postgenae rounded laterally; antennomeres IX–XI enlarged; antennomeres IX modified in male; pronotum rounded at anterolateral margins; male with broad metaventral processes; metacoxae simple; aedeagus with asymmetric median lobe.

###### Description.

**Figure 12.**
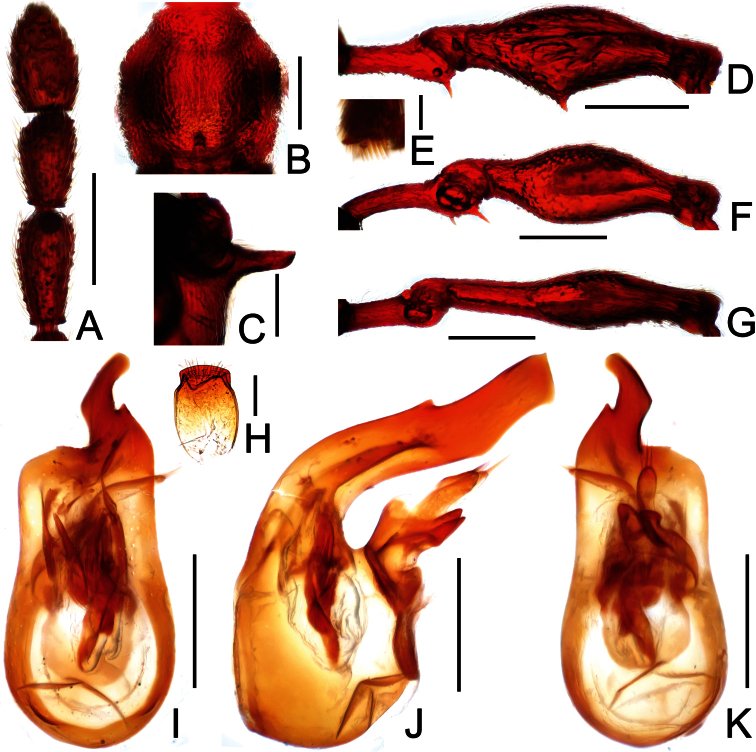
Diagnostic features of *Pselaphodes hainanensis* in male. **A** antenna **B** pronotum **C** median metaventral process, in lateral view **D** protrochanter and profemur **E** apical portion of protibia **F** mesotrochanter and mesofemur **G** metatrochanter and metafemur **H** sternite IX **I** aedeagus, in dorsal view **J** same, in lateral view **K** same, in ventral view. Scales (mm): **A, B, D, F, G** = 0.3; **C, I, J, K** = 0.2; **H** = 0.1; **E** = 0.05.

Male (Fig. 10B). Length 3.14–3.33. Head longer than wide, HL 0.69–0.72, HW 0.63–0.65; eyes each composed of about 40 facets. Antennal clubs as in Fig. 12A. Pronotum (Fig. 12B) slightly longer than wide, PL 0.65–0.68, PW 0.63–0.65, rounded at anterolateral margins. Elytra wider than long, EL 0.95–1.00, EW 1.28–1.32. Metaventral processes broad, apically narrowed (Fig. 12C). Protrochanters and profemora spinose ventrally (Fig. 12D), protibiae with tiny apical spur (Fig. 12E); mesotrochanters with two ventral spines, mesofemora simple (Fig. 12F); metatrochanters and metafemora simple (Fig. 12G). Abdomen broad at base and narrowed apically, AL 0.85–0.93, AW 1.26–1.31. Sternite IX as in Fig. 12H. Aedeagus length 0.61, with asymmetric median lobe (Figs 12I–K).

Female. Similar to male in general; BL 3.28–3.43, HL 0.75–0.80, HW 0.62–0.69, PL 0.71–0.72, PW 0.67–0.68, EL 0.87–0.93, EW 1.28–1.35, AL 0.95–0.98, AW 1.35–1.46. Eyes each composed of about 30 facets. Antennae unmodified; metaventral processes absent.

###### Comparative notes.

This new species can be separated from the other members of the group primarily by the short, thick metaventral processes, the rather elongate and apically truncate median lobe of the aedeagus, the structure of the aedeagal endophallus, and its distribution.

###### Distribution.

South China: Hainan.

###### Biology.

Adults are commonly found in leaf litter of mixed forests.

###### Etymology.

The new species is named after the Province where the type locality lies.

##### Pselaphodes kuankuoshuiensis

Yin & Li sp. n.

urn:lsid:zoobank.org:act:0D35EE60-F1BC-4E61-9593-5EC12A8BB625

http://species-id.net/wiki/Pselaphodes_kuankuoshuiensis

Figs 13A 14

###### Type material

(2 ♂♂, 3 ♀♀)**.** Holotype: ♂, labeled ‘China: Guizhou Prov. / Suiyang County / Kuankuoshui N. R. / Baishaogou, 750–900 m / 2010.VI.05, Yin & Zhai leg.’ (SNUC) Paratypes: 1 ♂, 2 ♀♀, same label data as holotype (SNUC); 1 ♀, same label data, except ‘2010.VI.03, Lu, Yin & Zhai leg.’ (SNUC).

###### Diagnosis.

Reddish brown; length 2.99–3.27; postgenae rounded laterally; antennomeres IX–XI enlarged; antennomeres IX modified in male; pronotum rounded at anterolateral margins; male with short metaventral processes; metacoxae simple; aedeagus with asymmetric median lobe.

###### Description.

**Figure 13.**
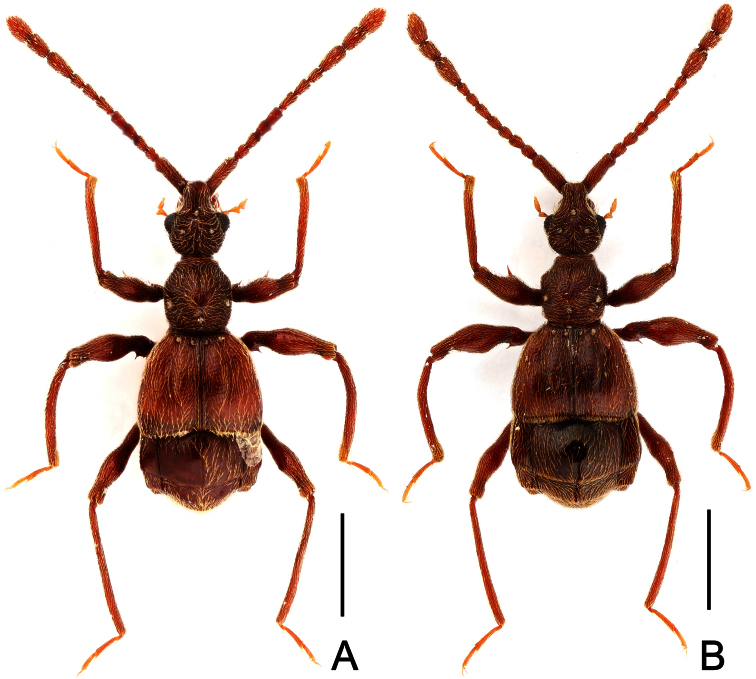
Male habitus of *Pselaphodes kuankuoshuiensis* (**A**) and *Pselaphodes longilobus* (**B**). Scales: 1.0 mm.

**Figure 14.**
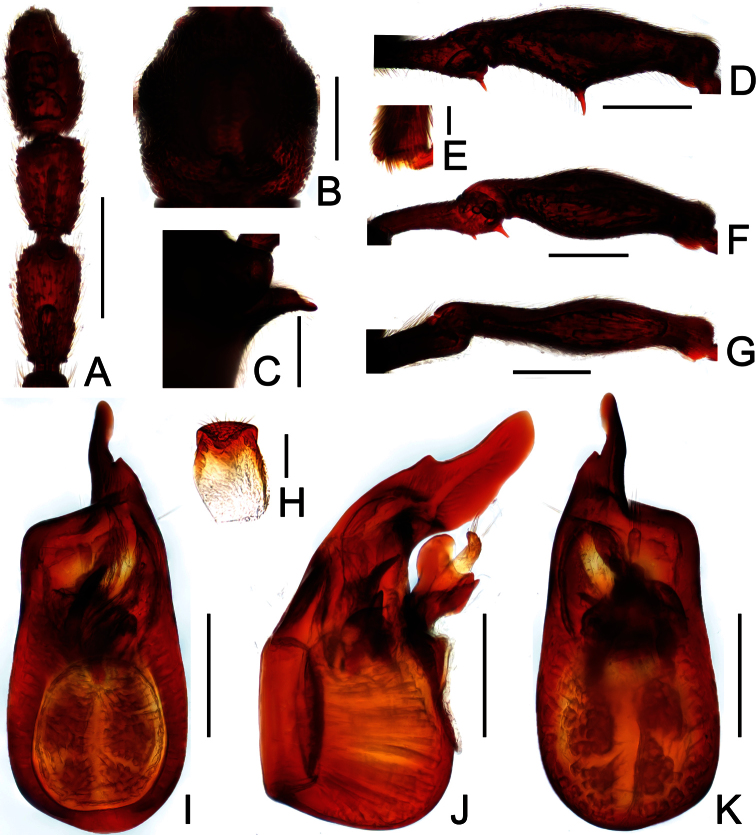
Diagnostic features of *Pselaphodes kuankuoshuiensis* in male. **A** antenna **B** pronotum **C** median metaventral process, in lateral view **D** protrochanter and profemur **E** apical portion of protibia **F** mesotrochanter and mesofemur **G** metatrochanter and metafemur **H** sternite IX **I** aedeagus, in dorsal view **J** same, in lateral view **K** same, in ventral view. Scales (mm): **A, B, D, F, G** = 0.3; **C, I, J, K** = 0.2; **H** = 0.1; **E** = 0.05.

Male (Fig. 13A). Length 2.99–3.12. Head longer than wide, HL 0.71–0.72, HW 0.64–0.65; eyes each composed of about 40 facets. Antennal clubs as in Fig. 14A. Pronotum (Fig. 14B) slightly longer than wide, PL 0.68–0.71, PW 0.63–0.65, rounded at anterolateral margins. Elytra wider than long, EL 0.90–0.92, EW 1.23–1.25. Metaventral processes short, apically narrowed (Fig. 14C). Protrochanters and profemora spinose ventrally (Fig. 14D), protibiae with small apical projection (Fig. 14E); mesotrochanters with two ventral spines, mesofemora simple (Fig. 14F); metatrochanters and metafemora simple (Fig. 14G). Abdomen broad at base and narrowed apically, AL 0.70–0.77, AW 1.20–1.22. Sternite IX as in Fig. 14H. Aedeagus length 0.61, with asymmetric median lobe (Figs 14I–K).

Female. Similar to male in general; BL 3.11–3.27, HL 0.71–0.73, HW 0.64–0.66, PL 0.70–0.71, PW 0.68–0.69, EL 0.90–0.95, EW 1.28–1.32, AL 0.80–0.88, AW 1.32–1.37. Eyes each composed of about 25 facets. Antennae unmodified; metaventral processes absent.

###### Comparative notes.

This species can be separated from the other members of the group by the short, apically narrowed metaventral processes, the apically rounded median lobe of the aedeagus, the structure of the aedeagal endophallus, and its distribution.

###### Distribution.

Southwest China: Guizhou.

###### Biology.

Adults were sifted from leaf litter along a road in a forest.

###### Etymology.

The new species is named after the type locality, Kuankuoshui Natural Reserve.

##### Pselaphodes longilobus

Yin & Hlaváč sp. n.

urn:lsid:zoobank.org:act:E0C29199-4328-468A-BE80-E4A231663A11

http://species-id.net/wiki/Pselaphodes_longilobus

Figs 13B 15

###### Type material

(4 ♂♂, 5 ♀♀)**.** Holotype: ♂, labeled ‘P. R. CHINA, Yunnan / Jizushan, 25°58'39"N, 100°21'14E / 28.VI.2011, 3216 m / sift27, V. Grebennikov’ (pcPH); Paratypes: 1 ♂, 4 ♀♀, same label data as holotype, except ‘25°58'18"N, 100°21'33N / 30.VI.2011, 2875 m / sift30’ (pcPH, SNUC); 2 ♂♂, 1 ♀, labeled ‘P. R. China, Hubei / Dabieshan, 31°06.013'N, 115°47.300'E / 11–21.VI.2008, 640 m / sifting, V. Grebennikov’ (pcPH, SNUC).

###### Diagnosis.

Reddish brown; length 3.31–3.37; postgenae rounded laterally; antennomeres IX–XI enlarged; antennomeres IX modified in male; pronotum rounded at anterolateral margins; male with long metaventral processes; metacoxae simple; aedeagus with asymmetric median lobe.

###### Description.

**Figure 15.**
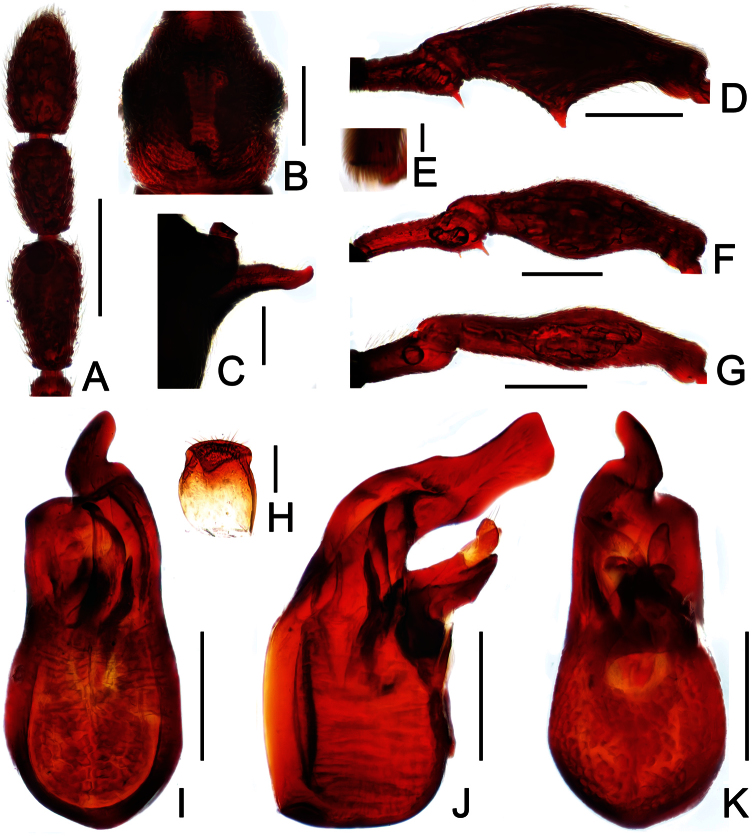
Diagnostic features of *Pselaphodes longilobus* in male. **A** antenna **B** pronotum **C** median metaventral process, in lateral view **D** protrochanter and profemur **E** apical portion of protibia **F** mesotrochanter and mesofemur **G** metatrochanter and metafemur **H** sternite IX **I** aedeagus, in dorsal view **J** same, in lateral view **K** same, in ventral view. Scales (mm): **A, B, D, F, G** = 0.3; **C, I, J, K** = 0.2; **H** = 0.1; **E** = 0.05.

Male (Fig. 13B). Length 3.31–3.37. Head longer than wide, HL 0.71–0.73, HW 0.63–0.64; eyes each composed of about 25 facets. Antennal clubs as in Fig. 15A. Pronotum (Fig. 15B) slightly longer than wide, PL 0.69–0.71, PW 0.61–0.66, rounded at anterolateral margins. Elytra wider than long, EL 0.87–0.88, EW 1.23–1.29. Metaventral processes broad and long, apically narrowed (Fig. 15C). Protrochanters and profemora spinose ventrally (Fig. 15D), protibiae with tiny apical projection (Fig. 15E); mesotrochanters with two ventral spines, mesofemora simple (Fig. 15F); metatrochanters and metafemora simple (Fig. 15G). Abdomen broad at base and narrowed apically, AL 1.04–1.05, AW 1.28–1.30. Sternite IX as in Fig. 15H. Aedeagus length 0.70, with asymmetric median lobe (Figs 15I–K).

Female. Similar to male in general; BL 3.36, HL 0.74, HW 0.61, PL 0.70, PW 0.65, EL 0.85, EW 1.29, AL 1.07, AW 1.38. Eyes each composed of about 20 facets. Antennae unmodified; metaventral processes absent.

###### Comparative notes.

This new species can be separated from the other species of the group by the metaventral processes being curved from the mid-length and then narrowed apically, the aedeagus with an elongate and apically truncate median lobe, the structure of the aedeagal endophallus, and its distribution.

###### Distribution.

Southwest China: Yunnan; Central China: Hubei.

###### Biology.

Individuals were sifted from leaf litter in forests.

###### Etymology.

The specific name refers to the long aedeagal median lobe of the new species.

##### Pselaphodes tianmuensis

Yin, Li & Zhao

http://species-id.net/wiki/Pselaphodes_tianmuensis

Figs 16A 17

Pselaphodes tianmuensis Yin, Li & Zhao, 2010: 22. Type locality: Tianmushan Mountain, Zhejiang, East China.
Pselaphodes wuyinus Yin, Li & Zhao, 2010: 23. Type locality: Wuyishan Mountain, Fujian, East China.

###### Type material examined.

[*Pselaphodes tianmuensis*] Holotype: ♂, labeled ‘CHINA: Zhejiang Prov. / West Tianmushan Mt. / 17.v.2006, alt. 300 m / HU & TANG leg.’ (SNUC). [*Pselaphodes wuyinus*] Holotype: ♂, labeled ‘CHINA: Fujian Prov. / Wuyishan Mt. / Tongmu Villege / 28.vii.2008, alt. 800 m / QI & YIN leg.’(SNUC).

###### Additional material examined.

1 ♂, 8 ♀♀, labeled ‘CHINA: Anhui Prov. / Guniujiang N. R. / 29.iv.2005, alt. 320–380 m / HU & TANG leg.’; 1 ♂, 3 ♀♀, labeled ‘CHINA: Guangxi Prov. / Jinxiu County / Laoshan, 7 km / 21.vii.2011, 1200–1400 m / J. Y. Hu & Z. W. Yin leg.’ (all SNUC).

###### Diagnosis and description.

Yin, Li and Zhao, 2010 (P22, figs 7, 19, 37, 38, 64, 65, 96, 114, 115, 133, 144, 162, 163, 181); Figs 16A, 17.

###### Distribution.

East China: Zhejiang, Anhui, Fujian, Jiagnxi; South China: Guangxi (**new provincial record**).

###### Comparative notes.

The *Pselaphodes tianmuensis* group is based on this species. *Pselaphodes tianmuensis* can be separated from the other members of the group by the short, apically narrowed metaventral processes combined with the apically rounded median lobe of the aedeagus, the structure of the aedeagal endophallus, and its distribution.

###### Notes.

The structure of aedeagal endophallus varies slightly among the populations from the listed localities. At this time we are not able to separate these populations at the species level.

**Figure 16.**
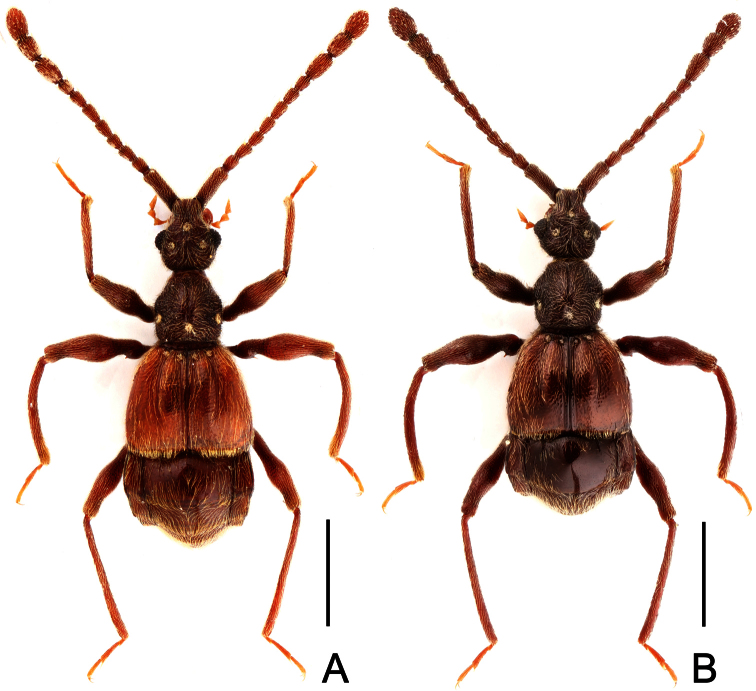
Male habitus of *Pselaphodes tianmuensis* (**A**) and *Pselaphodes tiantongensis* (**B**). Scales: 1.0 mm.

**Figure 17.**
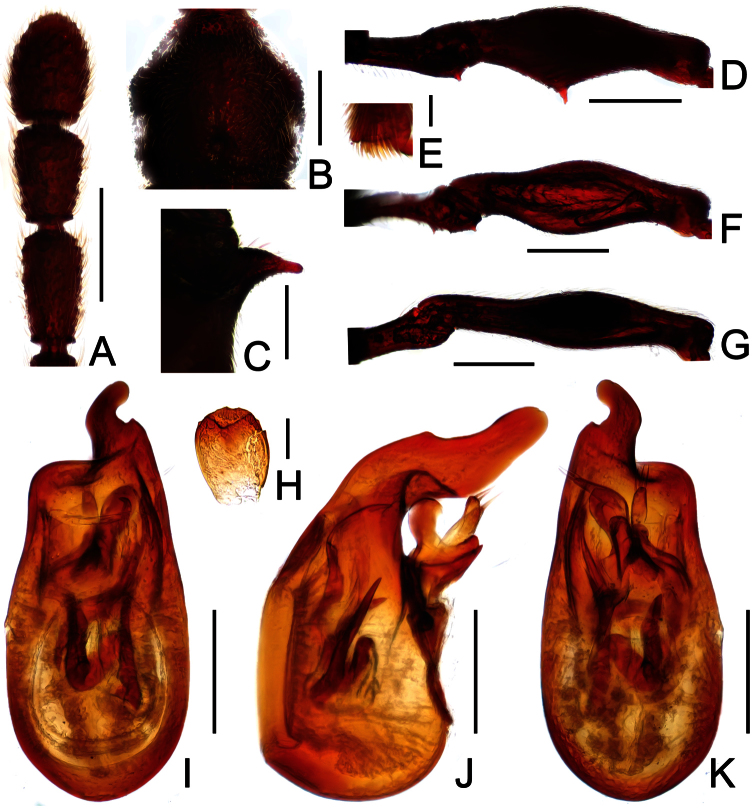
Diagnostic features of *Pselaphodes tianmuensis* in male. **A** antenna **B** pronotum **C** median metaventral process, in lateral view **D** protrochanter and profemur **E** apical portion of protibia **F** mesotrochanter and mesofemur **G** metatrochanter and metafemur **H** sternite IX **I** aedeagus, in dorsal view **J** same, in lateral view **K** same, in ventral view. Scales (mm): **A, B, D, F, G** = 0.3; **C, I, J, K** = 0.2; **H** = 0.1; **E** = 0.05.

##### Pselaphodes tiantongensis

Yin & Li sp. n.

urn:lsid:zoobank.org:act:ADE67E9B-9B6A-4BCD-ADC3-98DDAC2C4EB3

http://species-id.net/wiki/Pselaphodes_tiantongensis

Figs 16B 18

###### Type material

(5 ♂♂, 2 ♀♀)**.** Holotype: ♂, labeled ‘CHINA: Zhejiang, Ningbo City / Yinzhou District, Tiantong Shan / 29°48'03"N, 121°46'56E, 600 m / (mixed leaf litter, sifted) / 2009.iv.26, Ting Feng leg.’ (SNUC). Paratypes: 4 ♂♂, 2 ♀♀, same label data as holotype (SNUC).

###### Diagnosis.

Reddish brown; length 3.28–3.45; postgenae rounded laterally; antennomeres IX–XI enlarged; antennomeres IX modified in male; pronotum rounded at anterolateral margins; male with short metaventral processes; metacoxae simple; aedeagus with asymmetric median lobe.

###### Description.

**Figure 18.**
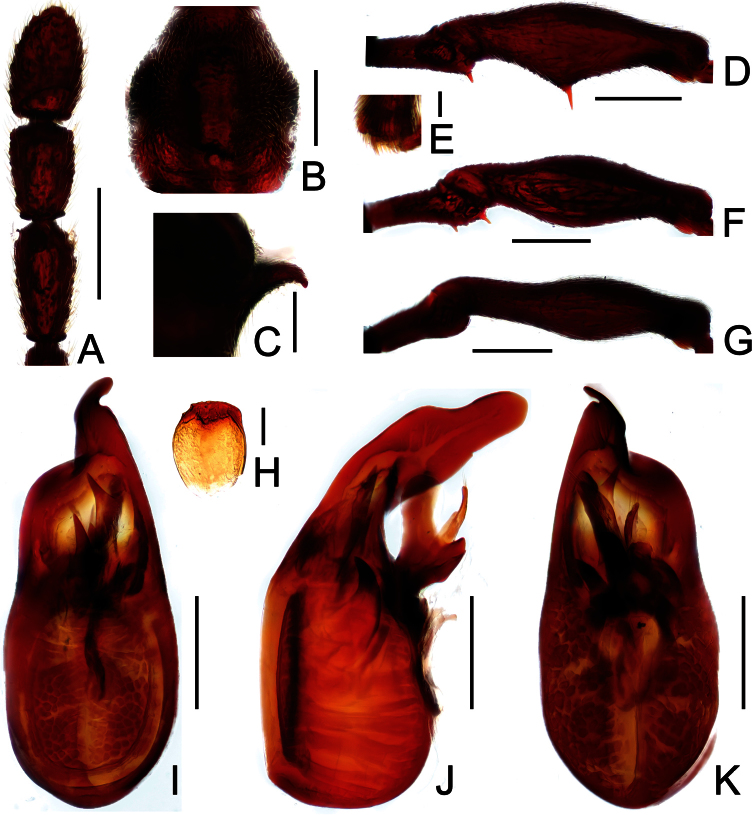
Diagnostic features of *Pselaphodes tiantongensis* in male. **A** antenna **B** pronotum **C** median metaventral process, in lateral view **D** protrochanter and profemur **E** apical portion of protibia **F** mesotrochanter and mesofemur **G** metatrochanter and metafemur **H** sternite IX **I** aedeagus, in dorsal view **J** same, in lateral view **K** same, in ventral view. Scales (mm): **A, B, D, F, G** = 0.3; **C, I, J, K** = 0.2; **H** = 0.1; **E** = 0.05.

Male (Fig. 16B). Length 3.34–3.45. Head longer than wide, HL 0.75–0.77, HW 0.67–0.68; eyes each composed of about 35 facets. Antennal clubs as in Fig. 18A. Pronotum (Fig. 18B) slightly longer than wide, PL 0.71–0.72, PW 0.66–0.69, rounded at anterolateral margins. Elytra wider than long, EL 1.00–1.01, EW 1.29–1.34. Metaventral processes short, apically narrowed and curved posteriorly (Fig. 18C). Protrochanters and profemora spinose ventrally (Fig. 18D), protibiae with indistinct apical projection (Fig. 18E); mesotrochanters with multiple ventral spines, mesofemora simple (Fig. 18F); metatrochanters and metafemora simple (Fig. 18G). Abdomen broad at base and narrowed apically, AL 0.88–0.95, AW 1.29–1.31. Sternite IX as in Fig. 18H. Aedeagus length 0.78, with asymmetric median lobe (Figs 18I–K).

Female. Similar to male in general; BL 3.28–3.37, HL 0.76–0.77, HW 0.63–0.66, PL 0.69–0.71, PW 0.69–0.70, EL 0.93–0.95, EW 1.29–1.35, AL 0.90–0.94, AW 1.34–1.40. Eyes each composed of about 30 facets. Antennae unmodified; metaventral processes absent.

###### Comparative notes.

This new species can be separated from the other species of the group by the short, apically curved and narrowed metaventral processes, the aedeagus with the median lobe being roundly broadened near apex, the structure of the aedeagal endophallus, and its distribution.

###### Distribution.

East China: Zhejiang.

###### Biology.

Individuals were sifted from mixed leaf litter of a forest.

###### Etymology.

The new species is named after the type locality, Tiantongshan National Forest Park.

##### Pselaphodes wrasei

Yin & Li sp. n.

urn:lsid:zoobank.org:act:9C525AD1-4FCA-43A6-A70A-2A5A7D5655E0

http://species-id.net/wiki/Pselaphodes_wrasei

Figs 19A 20

###### Type material

(1 ♂, 5 ♀♀)**.** Holotype: ♂, labeled ‘CHINA (N-Yunnan) Zhongdian Co. / 36 km ESE Zhongdian, 3500–3550 m / 27°40'09"N, 100°01'05E (over grown / rock hillside with old mixed forest, / bamboo, dead wood, leaf litter) / 23–24.VIII.2003 Wrase [13]’ (pcMS); 4 ♀♀, same label data as holotype (pcMS); 1 ♀, same label data, except ’24.VIII.2003, M. Schülke’ (pcMS).

###### Diagnosis.

Reddish brown; length 3.27–3.32; postgenae rounded laterally; antennomeres IX–XI enlarged; antennomeres IX modified in male; pronotum rounded at anterolateral margins; male with long metaventral processes; metacoxae simple; aedeagus with asymmetric median lobe.

###### Description.

**Figure 19.**
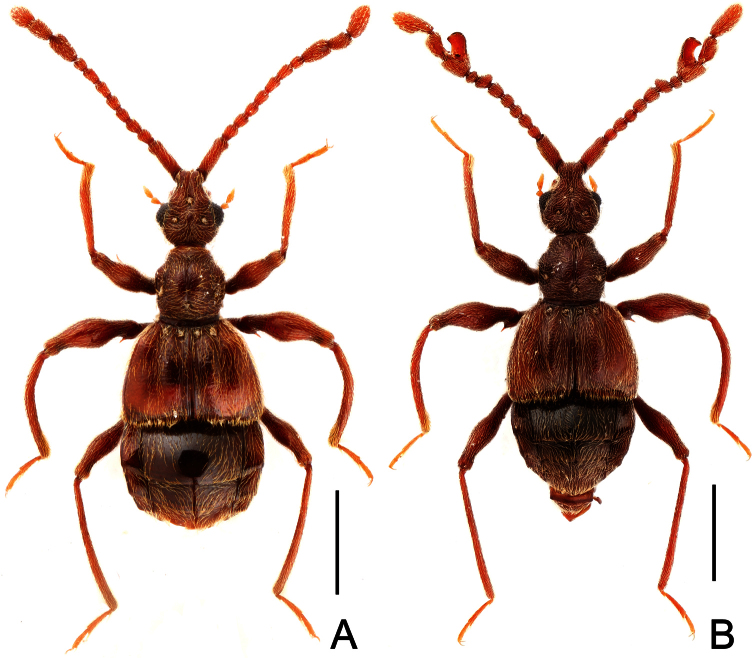
Male habitus of *Pselaphodes wrasei* (**A**) and *Pselaphodes grebennikovi* (**B**). Scales: 1.0 mm.

**Figure 20.**
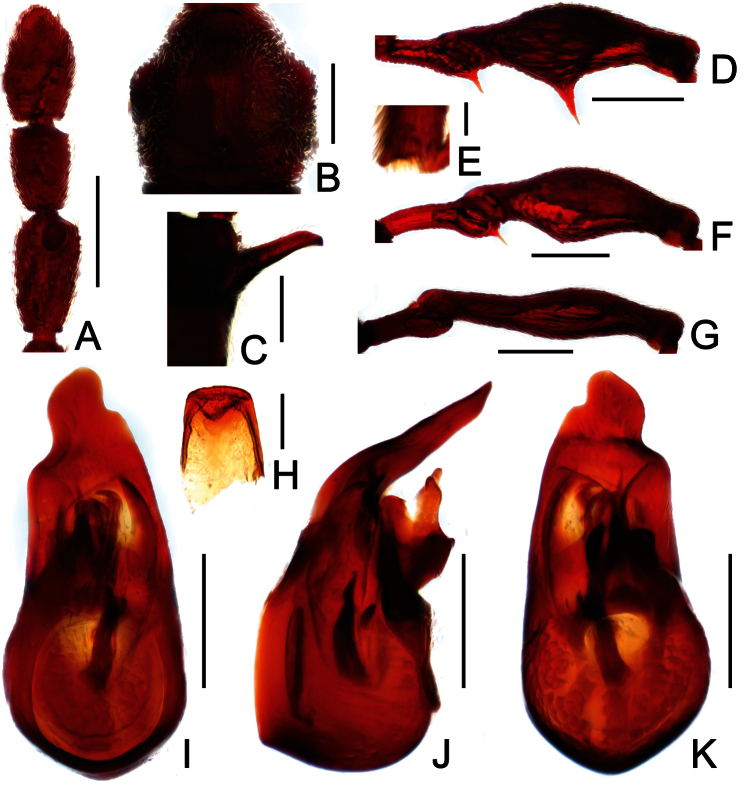
Diagnostic features of *Pselaphodes wrasei* in male. **A** antenna **B** pronotum **C** median metaventral process, in lateral view **D** protrochanter and profemur **E** apical portion of protibia **F** mesotrochanter and mesofemur **G** metatrochanter and metafemur **H** sternite IX **I** aedeagus, in dorsal view **J** same, in lateral view **K** same, in ventral view. Scales (mm): **A, B, D, F, G** = 0.3; **C, I, J, K** = 0.2; **H** = 0.1; **E** = 0.05.

Male (Fig. 19A). Length 3.32. Head longer than wide, HL 0.75, HW 0.68; eyes each composed of about 45 facets. Antennal clubs as in Fig. 20A. Pronotum (Fig. 20B) slightly longer than wide, PL 0.72, PW 0.69, rounded at anterolateral margins. Elytra wider than long, EL 0.89, EW 1.34. Metaventral processes long, apically narrowed (Fig. 20C). Protrochanters and profemora spinose ventrally (Fig. 20D), protibiae with small apical projection (Fig. 20E); mesotrochanters with single ventral spine, mesofemora simple (Fig. 20F); metatrochanters and metafemora simple (Fig. 20G). Abdomen broad at base and narrowed apically, AL 0.96, AW 1.41. Sternite IX as in Fig. 20H. Aedeagus length 0.62, with asymmetric median lobe (Figs 20I–K).

Female. Similar to male in general; BL 3.27–3.32, HL 0.73–0.74, HW 0.62–0.63, PL 0.68–0.70, PW 0.65–0.66, EL 0.81–0.82, EW 1.29–1.31, AL 1.05–1.06, AW 1.45–1.46. Eyes each composed of about 25 facets. Antennae unmodified; metaventral processes absent.

###### Comparative notes.

This species can be separated from the other species of the group by the thin, elongate metaventral processes, the thin median lobe of the aedeagus, the structure of the aedeagal endophallus, and its distribution.

###### Distribution.

Southwest China: Yunnan.

###### Biology.

Adults were collected by sifting leaf litter and moss in mixed forests.

###### Etymology.

The new species is named after David W. Wrase, collector of the holotype and most paratypes.

#### II. Other *Pselaphodes* species

##### Pselaphodes aculeus

Yin, Li & Zhao

http://species-id.net/wiki/Pselaphodes_aculeus

Pselaphodes aculeus Yin, Li & Zhao, 2010: 8. Type locality: Nabanhe Natural Reserve, Jinghong, Yunnan, Southwest China.

###### Additional material examined

(2 ♂♂, 4 ♀♀)**.** 1 ♂, 3 ♀♀, labeled ‘CHINA: FUJIAN Prov. / Wuyi Shan Nat. Res. / Sangan env. (900 m) / 3..V.-12.VI.2001 / Hlaváč & Cooter lgt.’ (pcPH); 1 ♂, labeled ‘Baigecunbian [near Baihe Village] / 400 m alt., Napo / Guangxi, CHINA / 8.iv.1998 / Hai-Sheng Zhou leg.’ (pcPH)

###### Diagnosis and description.

Yin, Li and Zhao, 2010 (P 8; figs 11, 23, 49–51, 68–70, 84, 85, 100, 122, 123, 136, 148, 170, 171, 177); Yin, Li and Zhao, 2011a (P 476; figs 111–116).

###### Distribution.

East China: Anhui, Fujian (**new provincial record**); Southwest China: Yunnan; South China: Guangxi (**new provincial record**), Hainan.

###### Comments.

The male pro- and metatibiae of this species are uniquely modified. Populations from different localities have the aedeagus differing in the apices of median lobe and endophallus. Since the male external features are quite stable, all populations are treated as one, wide-spread species.

##### Pselaphodes grebennikovi

Yin & Hlaváč sp. n.

urn:lsid:zoobank.org:act:C7FCE72A-D98C-49E7-B941-5FDFC1DED19E

http://species-id.net/wiki/Pselaphodes_grebennikovi

Figs 19B 21

###### Type material 

(2 ♂♂, 5 ♀♀)**.** Holotype: ♂, labeled ‘CHINA, YunnaN, Cang Shan at Dali / 25°41'07"N, 100°06'58E / 2.VII.2011, 2714 m / sift33. V. Grebennikov’ (pcPH). Paratypes: 1 ♂, 5 ♀♀, same label data as holotype (pcPH, SNUC).

###### Diagnosis.

Reddish brown; length 3.21–3.55; postgenae rounded laterally; antennomeres IX–XI enlarged; VII and IX–XI modified in male; pronotum rounded at anterolateral margins; male with long, broad metaventral processes; metacoxae simple; aedeagus with asymmetric median lobe.

###### Description.

**Figure 21.**
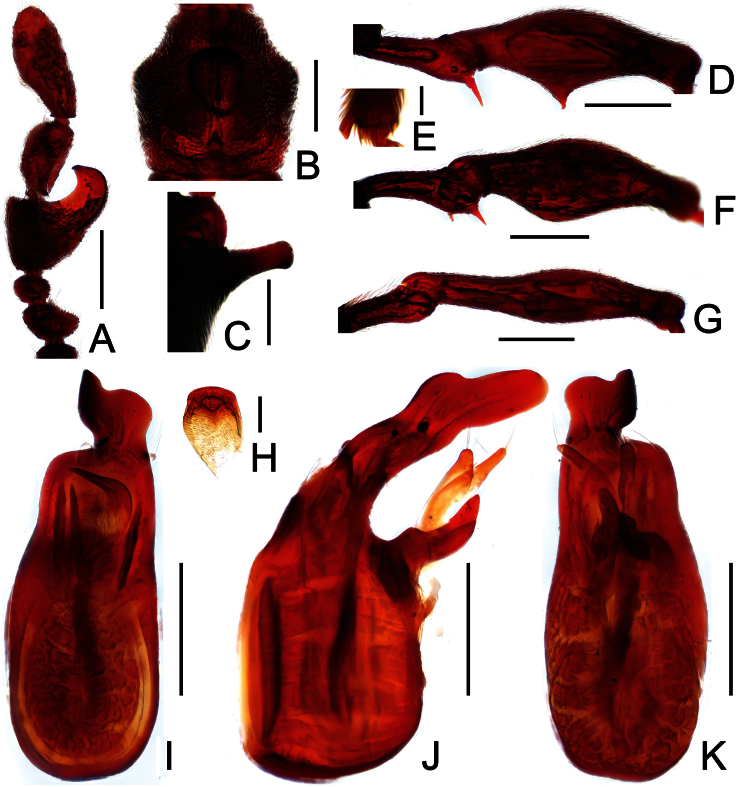
Diagnostic features of *Pselaphodes grebennikovi* in male. **A** antenna **B** pronotum **C** median metaventral process, in lateral view **D** protrochanter and profemur **E** apical portion of protibia **F** mesotrochanter and mesofemur **G** metatrochanter and metafemur **H** sternite IX **I** aedeagus, in dorsal view **J** same, in lateral view **K** same, in ventral view. Scales (mm): **A, B, D, F, G** = 0.3; **C, I, J, K** = 0.2; **H** = 0.1; **E** = 0.05.

Male (Fig. 19B). Length 3.37–3.55. Head longer than wide, HL 0.76–0.80, HW 0.66–0.69; eyes each composed of about 40 facets. Antennal clubs as in Fig. 21A. Pronotum (Fig. 21B) about as long as wide, PL 0.71–0.75, PW 0.71–0.73, rounded at anterolateral margins. Elytra wider than long, EL 0.90–0.93, EW 1.32–1.37. Metaventral processes long, and broad (Fig. 21C). Protrochanters and profemora spinose at ventral margins (Fig. 21D), protibiae with distinct blunt apical spur (Fig. 21E); mesotrochanters with small ventral spines, mesofemora simple (Fig. 21F); metatrochanters and metafemora simple (Fig. 21G). Abdomen broad at base and narrowed apically, AL 1.00–1.07, AW 1.29–1.38. Sternite IX as in Fig. 21H. Aedeagus length 0.57, with asymmetric median lobe (Figs 21I–K).

Female. Similar to male in general; BL 3.21–3.31, HL 0.74–0.75, HW 0.61–0.62, PL 0.71–0.72, PW 0.69–0.71, EL 0.83–0.84, EW 1.31–1.32, AL 0.93–1.00, AW 1.36–1.37. Eyes each composed of about 25 facets. Antennae unmodified; metaventral processes absent.

###### Comparative notes.

This distinct species can be readily separated from all other members of the genus by the antennomeres IX being largely projecting mesally, the modified antennomeres VII, and the aedeagus with a long, apically rounded median lobe.

###### Distribution.

Southwest China: Yunnan.

###### Biology.

Individuals were collected by sifting leaf litter in a forest.

###### Etymology.

The new species is named after Vasily Grebennikov, collector of the type series.

##### Pselaphodes maoershanus

Yin & Li

http://species-id.net/wiki/Pselaphodes_maoershanus

Figs 22A 23

Pselaphodes maoershanus Yin & Li, 2012 (Yin et al. 2012: 35). Type locality: Maoershan Mountain, Guilin, Guangxi, South China.

###### Additional material examined.

1 ♂, 2 ♀♀, labeled ‘CHINA: Guizhou, Leishan Co. / SE Kaili, NE Leishan / Leigong Shan, E-slope / 26°23'39"N, 108°13'33E // 2.5 km E of pass / 23–24.6.2001 / ca. 1600 m / leg. Schillhammer (17A)’ (pcPH).

###### Diagnosis and description.

Yin et al. 2012 (P35; figs 3, 6, 9, 12, 15, 18, 21, 24, 27, 30); figs 22A, 23.

###### Distribution.

South China: Guangxi; Southwest China: Guizhou (**new provincial record**).

###### Comments.

Adults from Leigongshan Mountain are readily identified as *Pselaphodes maoershanus* based on the male features being identical with those from the type locality.

**Figure 22.**
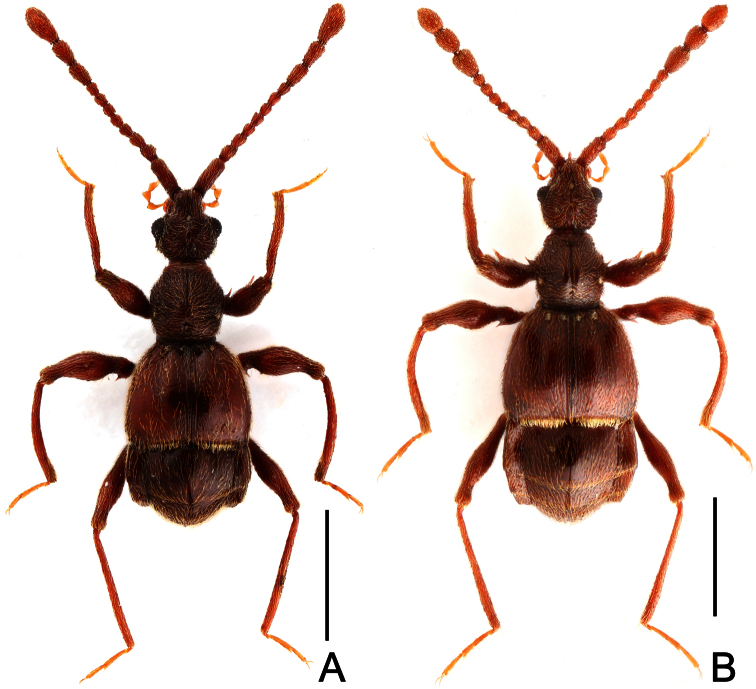
Male habitus of *Pselaphodes maoershanus* (**A**) and *Pselaphodes monoceros* (**B**). Scales: 1.0 mm.

**Figure 23.**
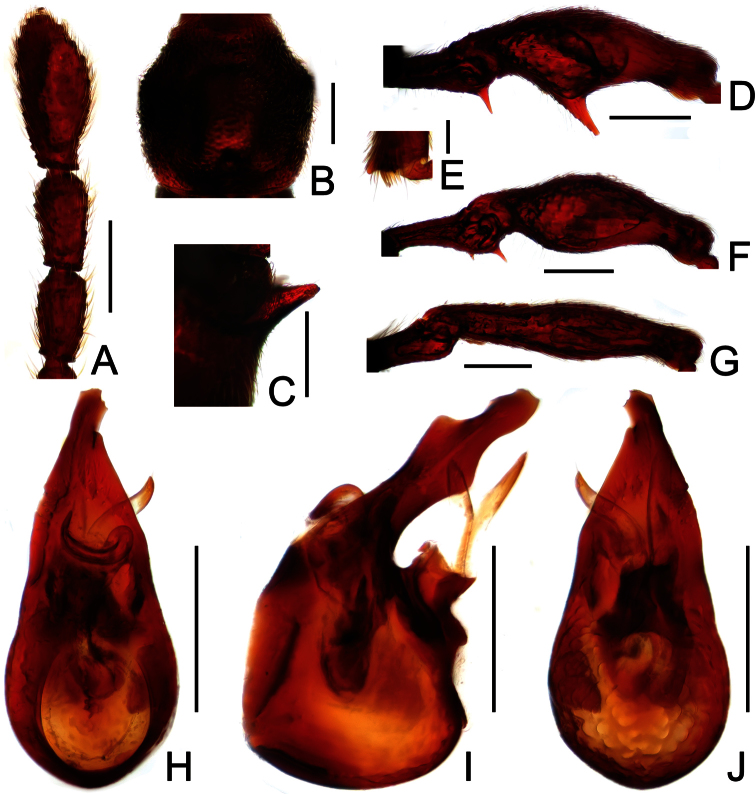
Diagnostic features of *Pselaphodes maoershanus* in male. **A** antenna **B** pronotum **C** median metaventral process, in lateral view **D** protrochanter and profemur **E** apical portion of protibia **F** mesotrochanter and mesofemur **G** metatrochanter and metafemur **H** aedeagus, in dorsal view **I** same, in lateral view **J** same, in ventral view. Scales (mm): all 0.2, except E = 0.05.

##### Pselaphodes monoceros

Yin & Hlaváč sp. n.

urn:lsid:zoobank.org:act:8A403224-2E7F-4422-802C-957017D73558

http://species-id.net/wiki/Pselaphodes_monoceros

Figs 22B 24

###### Type material

(5 ♂♂, 1 ♀)**.** Holotype: ♂, labeled ‘China: Xizang Prov. / Cuona County / Lexiang, alt. 2500 m / 16.vii.2012, Ye Liu leg.’ (SNUC); Paratypes: 4 ♂♂, 1 ♀, same label data as holotype type (SNUC).

###### Diagnosis.

Reddish brown; length 2.91–3.03; clypeus projected anteriorly, forming a horn-like process in male; postgenae elongate, rounded laterally; antennomeres IX–XI enlarged; pronotum rounded at anterolateral margins; male with greatly elongate metaventral processes; metacoxae simple; aedeagus with symmetric median lobe.

###### Description.

**Figure 24.**
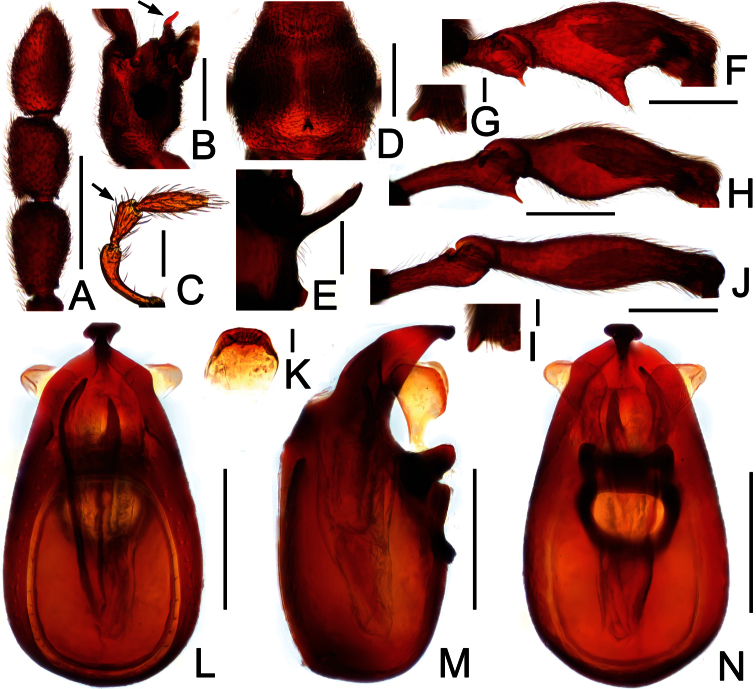
Diagnostic features of *Pselaphodes monoceros* in male. **A** antenna **B** head, in lateral view **C** maxillary palpus **D** pronotum **E** median metaventral process, in lateral view **F** protrochanter and profemur **G** apical portion of protibia **H** mesotrochanter and mesofemur **I** apical portion of mesotibia **J** metatrochanter and metafemur **K** sternite IX **L** aedeagus, in dorsal view **M** same, in lateral view **N** same, in ventral view. Scales (mm): **A, B, D, F, H, J** = 0.3; **E, L, M, N** = 0.2; **C** = 0.1; **G, I, K** = 0.05.

Male (Fig. 22B). Length 2.91–3.00. Head slightly longer than wide, HL 0.58–0.59, HW 0.56–0.58; clypeus projecting anteriorly (Fig. 24B); maxillary palpi (Fig. 24C) with segments III indistinctly projected laterally; eyes each composed of about 40 facets. Antennal clubs as in Fig. 24A. Pronotum (Fig. 24D) slightly longer than wide, PL 0.58–0.61, PW 0.55–0.59, rounded at anterolateral margins. Elytra wider than long, EL 0.89–0.90, EW 1.16–1.17. Metaventral processes greatly elongate, apically narrowed (Fig. 24E). Protrochantersand profemora spinose ventrally (Fig. 24F), protibiae with small apical spur (Fig. 24G); mesotrochanters spinose ventrally, mesofemora simple (Fig. 24H), mesotibiae with small apical spine (Fig. 24I); metatrochanters and metafemora simple (Fig. 24J). Abdomen broad at base and narrowed apically, AL 0.86–0.90, AW 1.16–1.19. Sternite IX as in Fig. 24K. Aedeagus length 0.56, with symmetric median lobe (Figs 24L–N).

Female. Similar to male in general; BL 3.03, HL 0.62, HW 0.57, PL 0.62, PW 0.60, EL 0.7, EW 1.19, AL 1.06, AW 1.28. Eyes each composed of about 20 facets. Antennae unmodified; metaventral processes absent.

###### Comparative notes.

This unusual *Pselaphodes* species has simple maxillary palpomeres II and IV, with palpomeres III only slightly projecting laterally on the anterolateral margins. This form of maxillary palpi together with the modified clypeus in the male is not known in any other species of the *Pselaphodes* complex of genera. These two characters, in combination with the form of the antennal clubs, and the greatly elongate metaventral processes readily separate the new species from all other congeners of the genus. The generic limit of *Pselaphodes* is expanded based on this species. The form of maxillary palpi seems to be occasionally variable within genus (also see comments on *Labomimus simplicipalpus* above). An extensive species-level phylogenetic analysis is needed for the determination of the taxonomic placements of these atypical species.

###### Distribution.

Southwest China: Xizang (= Tibet).

###### Biology.

Adults were collected by beating a pile of mixed live and dead branches in a forest.

###### Etymology.

The Latin word ‘*monoceros*’ means ‘a unicorn’, referring to the unique protuberance on the clypeus in the male.

##### Pselaphodes pectinatus

Yin, Li & Zhao

http://species-id.net/wiki/Pselaphodes_pectinatus

Figs 25A 26

Pselaphodes pectinatus Yin, Li & Zhao, 2011a: 474. Type locality: Bawangling Natural Reserve, Changjiang, Hainan, South China.

###### Additional material examined.

1 ♂, labeled ‘China: Hainan Prov. / Wuzhishan Mt. / road to peak / 18.iv.2012, 650–700 m / Peng et al. leg.’ (SNUC).

###### Diagnosis and description.

**Figure 25.**
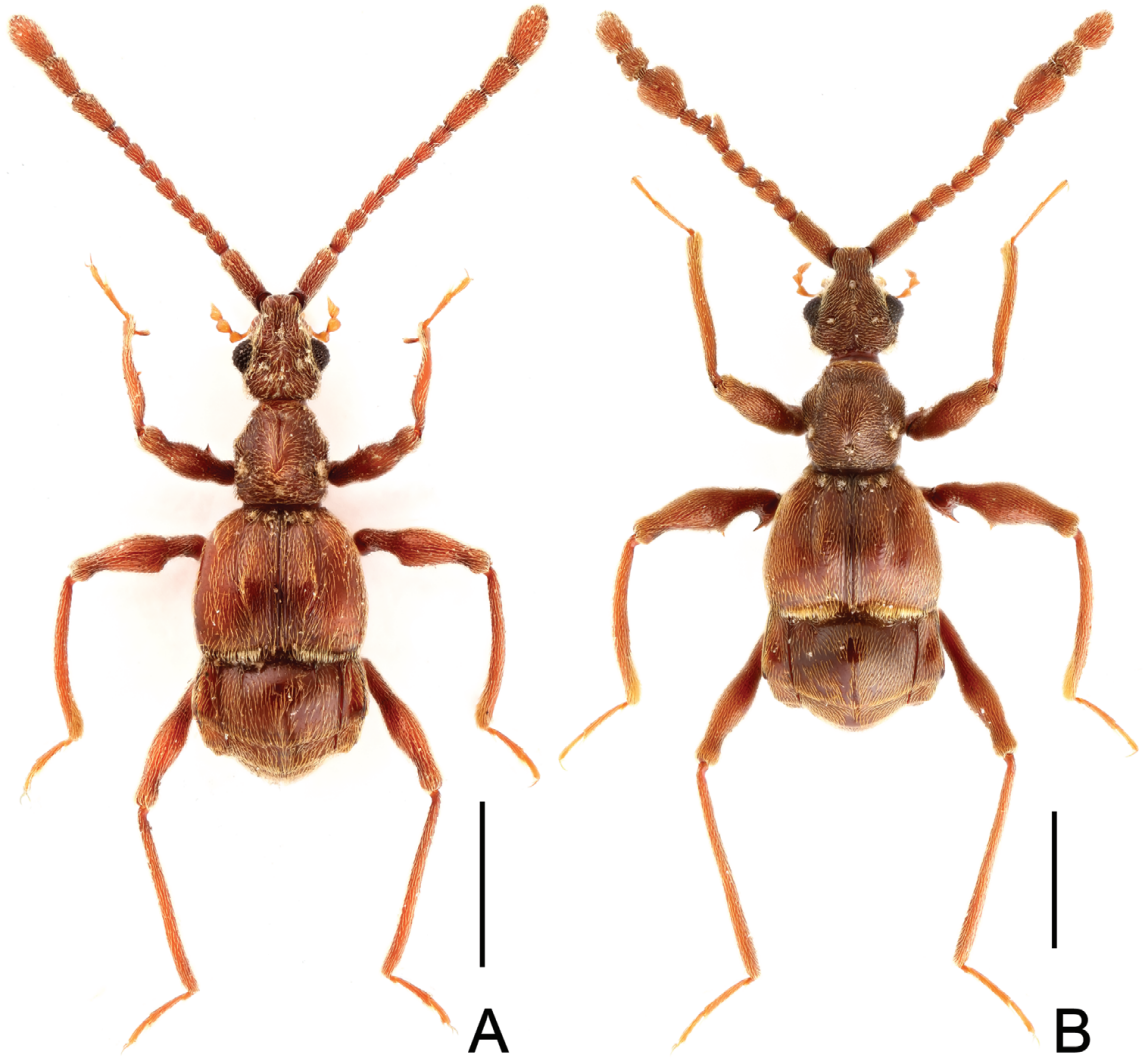
Male habitus of *Pselaphodes pectinatus* (**A**) and *Pselaphodes pengi* (**B**). Scales: 1.0 mm.

**Figure 26.**
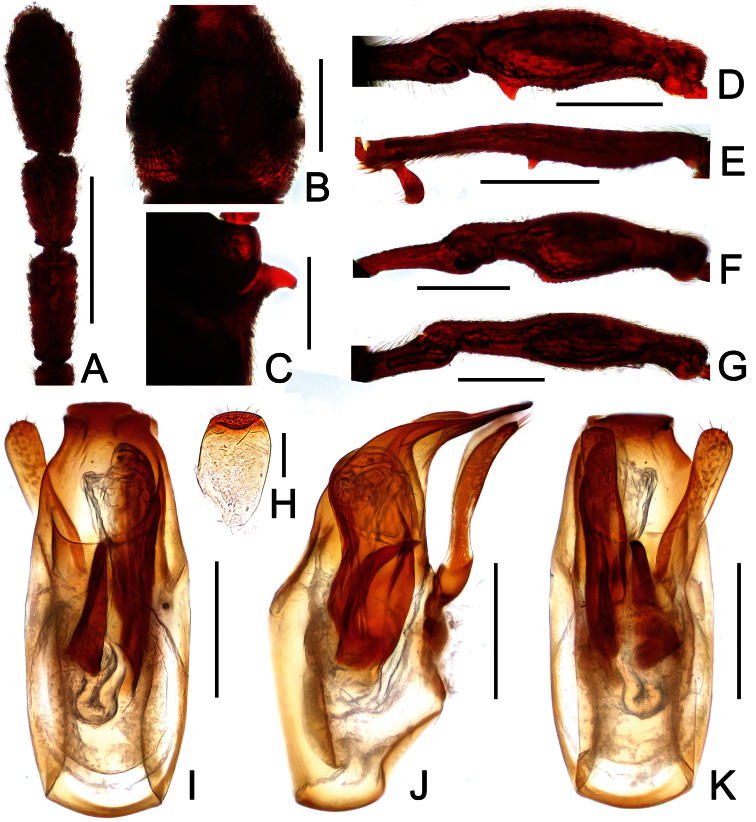
Diagnostic features of *Pselaphodes pectinatus* in male. **A** antenna **B** pronotum **C** median metaventral process, in lateral view **D** protrochanter and profemur **E** protibia **F** mesotrochanter and mesofemur **G** metatrochanter and metafemur **H** sternite IX **I** aedeagus, in dorsal view **J** same, in lateral view **K** same, in ventral view. Scales (mm): **A, B, D, E F, G** = 0.3; **C, I, J, K** = 0.2; **H** = 0.1.

Yin et al. 2011a (P474; figs 3 11, 23, 35, 47, 59, 63, 76, 89); figs 25A, 26.

###### Distribution.

South China: Hainan.

###### Comments.

This species was described from a single male from Bawangling, Hainan. The aedeagus of the holotype was lost during the dissection. Here we provide new illustrations of major diagnostic features of this species including the aedeagus, based on a second male specimen from Wuzhishan Mountain, Hainan. *Pselaphodes pectinatus* can be readily separated from all other congeners at the first sight by the greatly modified apical portion of the protibiae in the male.

##### Pselaphodes pengi

Yin & Li sp. n.

urn:lsid:zoobank.org:act:BEDE3E50-7062-420D-9320-971586BF0B10

http://species-id.net/wiki/Pselaphodes_pengi

Figs 25B 27

###### Type material

(3 ♂♂)**.** Holotype: ♂, labeled ‘CHINA: Sichuan, Tianquan County / Labahe N. R., Heixuan Valley, ca. 30 / km NW Tianquan, 30°10'36"N, 102°28'04E, 2000 m, (mixed leaf litter / sifted), 2012.vii.10, Dai, Peng, Yin’ (SNUC). Paratypes: 1 ♂, same label data as holotype (SNUC); 1 ♂, labeled ‘CHINA: Sichuan, E’meishan City / E’mei Shan Mt., pass between / Jiuling Hill and Xixinsuo Temple / 29°33'15"N, 103°21'24E, 1800 m / (leaf litter, sifted), 2012.vii.24 / C. C. Dai, Z. Peng & Z. W. Yin leg.’ (SNUC).

###### Diagnosis. 

Reddish brown; length 3.41–3.50; postgenae rounded laterally; antennomeres IX–XI enlarged; VI–VII and IX–XI modified in male; pronotum rounded at anterolateral margins; male with long metaventral processes; metacoxae simple; aedeagus with asymmetric median lobe.

###### Description.

**Figure 27.**
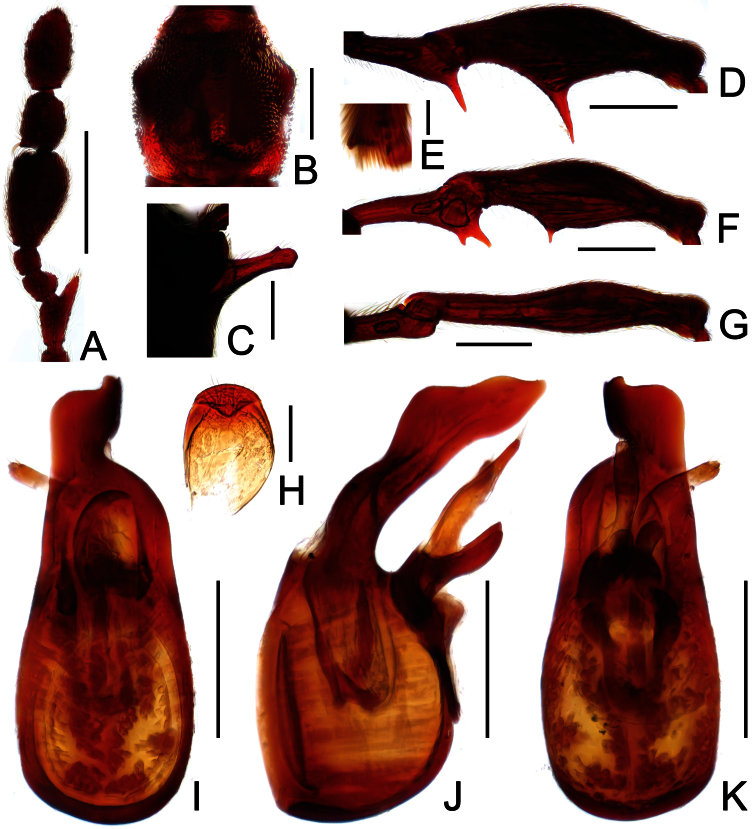
Diagnostic features of *Pselaphodes pengi* in male. **A** antenna **B** pronotum **C** median metaventral process, in lateral view **D** protrochanter and profemur **E** apical portion of protibia **F** mesotrochanter and mesofemur **G** metatrochanter and metafemur **H** sternite IX **I** aedeagus, in dorsal view **J** same, in lateral view **K** same, in ventral view. Scales (mm): **A, B, D, F, G** = 0.3; **C, I, J, K** = 0.2; **H** = 0.1; **E** = 0.05.

Male (Fig. 25B). Length 3.41–3.50. Head longer than wide, HL 0.76–0.78, HW 0.74–0.75; eyes each composed of about 50 facets. Antennal clubs as in Fig. 27A. Pronotum (Fig. 27B) slightly longer than wide, PL 0.78–0.79, PW 0.74–0.75, rounded at anterolateral margins. Elytra wider than long, EL 0.94–0.99, EW 1.32–1.35. Metaventral processes long, apically broadened (Fig. 27C). Protrochanters and profemora strongly spinose at ventral margins (Fig. 27D), protibiae with small apical spur (Fig. 27E); mesotrochanters with distinct ventral spines, mesofemora with small ventral spine (Fig. 27F); metatrochanters and metafemora simple (Fig. 27G). Abdomen broad at base and narrowed apically, AL 0.93–0.94, AW 1.31–1.37. Sternite IX as in Fig. 27H. Aedeagus length 0.60, with asymmetric median lobe (Figs 27I–K).

Female. Unknown.

###### Comparative notes.

The new species has unique, modified antennomeres VI, combined with the slightly modified antennomeres VII, the enlarged antennomeres IX with a round apical process, the metaventral processes each with a preapical denticle on the upper surface, and the aedeagus with an apically greatly broadened median lobe, it can be quickly separated from all other species of the genus. Currently there is no other *Pselaphodes* species known to process modified antennomeres VI in the male.

###### Distribution.

Southwest China: Sichuan.

###### Biology.

Individuals were sifted from leaf litter along roads in forests.

###### Etymology.

This species is named after Zhong Peng, co-collector of the type series.

## Supplementary Material

XML Treatment for Dayao emeiensis

XML Treatment for Labomimus fimbriatus

XML Treatment for Labomimus jizuensis

XML Treatment for Labomimus sichuanicus

XML Treatment for Labomimus simplicipalpus

XML Treatment for Labomimus tibialis

XML Treatment for Labomimus venustus

XML Treatment for Pselaphodes anhuianus

XML Treatment for Pselaphodes daii

XML Treatment for Pselaphodes hainanensis

XML Treatment for Pselaphodes kuankuoshuiensis

XML Treatment for Pselaphodes longilobus

XML Treatment for Pselaphodes tianmuensis

XML Treatment for Pselaphodes tiantongensis

XML Treatment for Pselaphodes wrasei

XML Treatment for Pselaphodes aculeus

XML Treatment for Pselaphodes grebennikovi

XML Treatment for Pselaphodes maoershanus

XML Treatment for Pselaphodes monoceros

XML Treatment for Pselaphodes pectinatus

XML Treatment for Pselaphodes pengi
